# Semi-Supervised Clustering-Based DANA Algorithm for Data Gathering and Disease Detection in Healthcare Wireless Sensor Networks (WSN)

**DOI:** 10.3390/s24010018

**Published:** 2023-12-19

**Authors:** Anurag Sinha, Turki Aljrees, Saroj Kumar Pandey, Ankit Kumar, Pallab Banerjee, Biresh Kumar, Kamred Udham Singh, Teekam Singh, Pooja Jha

**Affiliations:** 1 Department of Computer Science and Information Technology, IIndira Gandhi National Open University, New Delhi 110068, India; anuragsinha257@gmail.com; 2 Department College of Computer Science and Engineering, University of Hafr Al-Batin, Hafar Al-Batin 39524, Saudi Arabia; tajrees@uhb.edu.sa; 3 Department of Computer Engineering & Applications, GLA University, Mathura 281406, India; saroj.pandey@gla.ac.in; 4 Department of Information Technology, Guru Ghasidas Vishwavidyalaya, Bilaspur 495001, India; 5 Department of Computer Science and Information Technology, Amity University Jharkhand, Ranchi 834001, India; pbanerjee@rnc.amity.edu (P.B.); bkumar@rnc.amity.edu (B.K.); pjha@rnc.amity.edu (P.J.); 6 School of Computing, Graphic Era Hill University, Dehradun 248002, India; 11004033@gs.ncku.edu.tw; 7 Department of Computer Science and Engineering, Graphic Era Deemed to Be University, Dehradun 248002, India; tsingh@ma.iitr.ac.in

**Keywords:** WSN, data gathering, data mining, KGS theory, signal processing, healthcare, clustering

## Abstract

Wireless sensor networks (WSNs) have emerged as a promising technology in healthcare, enabling continuous patient monitoring and early disease detection. This study introduces an innovative approach to WSN data collection tailored for disease detection through signal processing in healthcare scenarios. The proposed strategy leverages the DANA (data aggregation using neighborhood analysis) algorithm and a semi-supervised clustering-based model to enhance the precision and effectiveness of data collection in healthcare WSNs. The DANA algorithm optimizes energy consumption and prolongs sensor node lifetimes by dynamically adjusting communication routes based on the network’s real-time conditions. Additionally, the semi-supervised clustering model utilizes both labeled and unlabeled data to create a more robust and adaptable clustering technique. Through extensive simulations and practical deployments, our experimental assessments demonstrate the remarkable efficacy of the proposed method and model. We conducted a comparative analysis of data collection efficiency, energy utilization, and disease detection accuracy against conventional techniques, revealing significant improvements in data quality, energy efficiency, and rapid disease diagnosis. This combined approach of the DANA algorithm and the semi-supervised clustering-based model offers healthcare WSNs a compelling solution to enhance responsiveness and reliability in disease diagnosis through signal processing. This research contributes to the advancement of healthcare monitoring systems by offering a promising avenue for early diagnosis and improved patient care, ultimately transforming the landscape of healthcare through enhanced signal processing capabilities.

## 1. Introduction

Wireless sensor networks (WSNs) consist of a multitude of distributed devices, equipped with sensors, to monitor physical or environmental conditions. These devices, also known as nodes, collaboratively pass their data through the network to a main location or sink where the data can be observed and analyzed. The nodes in a WSN are generally small in size and are often deployed in hard-to-reach or hazardous environments. They are capable of sensing a wide range of phenomena including temperature, humidity, pressure, and motion. WSNs are used in various applications such as environmental monitoring, health care, military, and industrial automation [1].

WSNs represents a transformative technology that has permeated various domains due to their ability to remotely monitor and collect data across extensive areas. By integrating sensor nodes, these networks provide real-time insights into environmental conditions, physical phenomena, and systems operations. Sensor nodes in a WSN are equipped with sensors, processors, and wireless communication interfaces, enabling them to sense, process, and communicate data [2].

Applications of WSN are diverse and span across numerous fields including environmental monitoring, health care, industrial automation, and military operations. In environmental monitoring, WSNs contribute significantly to tracking air and water quality, observing wildlife, and monitoring soil conditions. In the healthcare sector, they assist in patient monitoring, ensuring timely administration of drugs, and tracking the availability of medical resources. In industrial settings, WSNs are crucial for monitoring the condition of machinery, managing inventory, and ensuring safety protocols are adhered to. Similarly, in military operations, WSNs play a vital role in surveillance, target tracking, and securing perimeters [3].

Despite their wide applicability, the deployment of WSNs comes with challenges, particularly in terms of energy consumption and network management. Sensor nodes are typically battery-powered and deployed in hard-to-reach areas, necessitating efficient energy management to prolong network life and ensure reliable performance.

One of the main issues with WSN implementation for healthcare applications is effective data collecting. When multiple sensors are spread out across a vast area, collecting and managing data becomes a difficult task. Conventional data collection methods are typically time- and resource-consuming and insufficient given how dynamic and resource-constrained WSNs. In several industries, including healthcare, WSNs have become a game-changing technology. Innovative applications, such as signal processing-based disease diagnosis, have been made possible by their capacity to monitor patients’ physiological data in real-time and non-invasively. In order to enhance patient outcomes and enable prompt action, early and accurate disease diagnosis is essential in healthcare [4]. Deploying WSNs in healthcare environments, however, poses special difficulties. Limited resources, such as energy, memory, and computing power, necessitate effective data collection techniques in order to extend network lifetime and guarantee dependable operation. Furthermore, reliably identifying disease patterns from sensor data can be difficult, particularly when dealing with complicated and irregular disease patterns [5].

In order to overcome these difficulties, this research suggests a unique strategy that integrates the data collection DANA (dynamic agent node allocation) algorithm with the disease detection semi-supervised clustering-based model in WSNs. Utilizing adaptive and dynamic routing while reducing energy use and communication overhead, the DANA algorithm maximizes the effectiveness of data collection [6]. A reliable method for disease identification is provided by the semi-supervised clustering-based model, which makes use of both labeled and unlabeled data. Even with a small number of labeled samples, the model can more accurately diagnose diseases by using unlabeled data to efficiently capture complicated disease patterns [7].

WSN are built from a large number of inexpensive, low-powered, and modestly located remote sensors that are dispersed around a geographic region to perform irregular activities, such screening a genuine oddity [8]. WSNs were named the top emerging technology by MIT Technology Review in 2004; today, they are successfully used for a variety of purposes, including surveillance (e.g., constant location sound or video perception), security (e.g., identification of toxic artificial materials or regular subject matter experts), penchant checking (e.g., environmental assessment of temperature, pressure, or mechanical vibration), home motorization, and military systems. Standard remote sensor centers and base stations known as sinks are shown in a typical WSN topography. The sink is often linked to a power source and set up for use. Small-size batteries that, in the majority of application cases, are problematic or even difficult to replace or recharge frequently provide a challenge to conventional far-off sensor centers, which are prepared to deliver processed or unprocessed distinct data to the sink for practical reasons [9].

Consequently, rather than various other distant contraptions (e.g., cellular phones, PDAs, and workstations), when in doubt, it is not typical to restore the energy given to a far-off sensor center point during the presentation of the WSN. Consequently, every sensor center point is supposed to work under very low power use conditions. Overall, it is quite important to consider catch, transmission, and guidance difficulties, or the data collecting methods that determine how typical sensors perform for receiving information and passing it on to the sink, to develop a considerably energy-powerful WSN. As a result, acquiring data is a WSN’s primary and most crucial capability [10].

Numerous clever strategies devised in the past do not scale well with the astonishing increase in the number of people living on the devices and should be adjusted, updated, or new innovative strategies are intended to adapt to this significant turn of events. Additionally, as technology develops, new entrances become available that can be applied in this particular situation. In this work, data collection in WSN and IoT relationships is examined from a cross-layer approach. We provide a few foundational achievements and examples that have outlined the planning for different long-term coming-about strategies. We primarily focus on current state-of-the-art research that takes use of WSNs’ adaptability, energy efficiency, functionality, and human-friendliness [11]. In light of the data collection approach employed, we have recently structured our data collection tactics. In any event, it is possible to categorize data collection techniques similarly when taking into account the factors that influence data acquisition. Four main kinds, in particular, stand out [9]: event-driven, time-driven, request-based, and mixed. In an event-driven arrangement, data are created whenever an important event happens, whereas time-driven class data are occasionally provided from the sink at regular intervals in the class referred to as request-based cluster, data are amassed by sink requests. Finally, the cross-variety technique combines elements of one or more of the above methods. All of the aforementioned orders were given with the full anticipation of assessing energy usage and dependability for reenactment purposes. In any WSN, each sensor center point requires power for data transmission and social occasions, which is a critical perspective that impacts the display of enormous data gathering. Distant sensor networks are packed using two frameworks: cover bunch additionally, intra bundle [12]. Distant sensor association can be used for various applications like atmospheric conditions gauging, home auto-machine, military applications, prosperity noticing security applications, region-based unmistakable confirmation, environmental uses, and distinguishing and noticing consequently ideal estimations to store and which strong frameworks are not available to store and sense these stream sensor data [11].

The suggested research proposes the DANA algorithm and a semi-supervised clustering-based model particularly for data collection in WSNs utilized for signal processing-based disease diagnosis in healthcare to overcome these difficulties. The challenging issue of tracking and detecting diseases in the context of healthcare is effectively addressed by this ground-breaking method, which combines sophisticated data collection methods with intelligent processing. The cornerstone of this study is the DANA algorithm, which offers an effective and flexible data aggregation strategy adapted to the special features of WSNs. DANA maximizes data gathering by dynamically modifying the routing patterns, cutting down on energy use, and extending network life. This method uses network circumstances, data relevancy, and sensor node locations to intelligently route and aggregate data, assuring that only pertinent information is sent to the base station [12].

The semi-supervised clustering-based model, which combines machine learning and data analytics, works in tandem with the DANA algorithm to provide additional benefits. With the use of signal processing techniques, this model makes use of the gathered data to group significant sensor nodes into meaningful clusters, enabling more precise disease diagnosis. This model improves the accuracy of anomaly detection by using both supervised and unsupervised learning, giving healthcare workers useful information and lowering the number of false positives.

This study’s importance stems from its potential to change the way healthcare is delivered. Healthcare providers may gain from real-time, data-driven disease diagnosis by smoothly integrating the semi-supervised clustering-based model and the DANA algorithm into WSNs. The benefits of this proactive strategy include enhanced patient care, early intervention, and significant cost savings. Additionally, this technology is flexible and expandable. We will go into more depth about the semi-supervised clustering-based model and the DANA algorithm in the parts that follow, as well as how they work together to create a comprehensive solution for data collection and disease detection in healthcare WSNs. We will illustrate the efficiency and dependability of this method via empirical analyses and case studies, laying the foundation for a more connected and wholesome future in healthcare [13].

(a) Network Architecture: In a WSN, nodes are connected in a way that they can communicate with each other wirelessly. The architecture of WSN is typically categorized into two types: flat and hierarchical. In a flat architecture, all nodes have the same functionality, whereas in a hierarchical architecture, nodes have different roles and responsibilities. Regardless of the architecture type, the primary goal of WSN is to gather data from its environment and transmit it to a sink or base station.
(b) Communication Protocols: The communication in WSNs is managed by various protocols that ensure data is transmitted efficiently and reliably. These protocols take into account the limited resources of the sensor nodes, such as their energy constraints, to optimize the network’s performance.
(c) Energy Consumption: One of the critical challenges in WSN is managing the energy consumption of the sensor nodes. Since these nodes are often battery-powered and deployed in inaccessible locations, it is crucial to optimize their energy usage to prolong the network’s lifespan. Various techniques and algorithms have been developed to address this issue, focusing on energy-efficient data transmission, data aggregation, and power management.
(d) Applications: WSNs have a wide range of applications, spanning across various fields. In environmental monitoring, they are used to collect data on air and water quality, soil conditions, and wildlife activity. In health care, they assist in patient monitoring and drug administration. In industrial settings, they contribute to machine health monitoring and inventory management. Additionally, WSNs play a significant role in military operations for surveillance, target tracking, and battlefield monitoring.

WSNs consist of distributed sensor nodes that collaboratively monitor and collect data from various environments. These networks have gained prominence due to their versatility, scalability, and ability to operate in inaccessible or harsh conditions. The sensor nodes in a WSN are typically equipped with sensing, processing, and communication capabilities, allowing them to capture data, process it, and transmit it to a central location for analysis. Despite their advantages, WSNs face several challenges that impact their efficiency and reliability [12]. Key challenges include limited energy resources, data redundancy, network management, and security concerns. The sensor nodes are usually battery-powered, necessitating energy-efficient operations to prolong network lifetime. Data redundancy arises when multiple nodes cover overlapping areas, leading to unnecessary data transmission and increased energy consumption. Efficient network management is crucial for optimizing data flow and ensuring robust performance, while security measures are necessary to protect the network from malicious attacks.

Building upon recent advancements in unsupervised non-intrusive load monitoring (NILM) algorithms, which utilize graph Laplacian regularization (GLR) [13] for superior performance, this paper introduces an innovative unsupervised method. This new approach is centered on creating an underlying graph that effectively captures the correlations within time-series data from smart meters. It features a unique variable-length data segmentation strategy for isolating potential events. Graph-theoretical approaches have been widely adopted in WSNs to address these challenges, providing a structured way to analyze and optimize network performance [14]. In these approaches, the network is represented as a graph where nodes represent sensor devices, and edges represent communication links between them. Various graph algorithms and methods are applied to solve problems related to routing, network connectivity, and resource allocation. For instance, minimum spanning tree algorithms are used to create energy-efficient routing paths, ensuring data is transmitted with the least energy consumption. Graph coloring techniques [15] are applied to address data redundancy and improve resource allocation, ensuring that overlapping nodes do not transmit data simultaneously, thereby reducing interference and energy consumption [16]. The recent surge in graph signal processing-based applications has opened new avenues for addressing WSN challenges. Graph signal processing (GSP) extends classical signal processing techniques to data residing on irregular graph structures, making it particularly suitable for WSNs where sensor nodes are distributed in an irregular manner. GSP-based approaches enable the analysis of signals on graphs, allowing for the efficient processing of data collected by sensor nodes. These approaches have been applied to various WSN applications including environmental monitoring, health care, and industrial automation. For example, in environmental monitoring, GSP-based techniques are used to analyze data collected from sensor nodes to detect anomalies and predict environmental conditions. In healthcare, they assist in processing data from wearable devices for patient monitoring and diagnosis.

The application of graph theoretical and graph signal processing-based approaches in WSNs provides a robust framework for addressing the network’s inherent challenges. These approaches enable the efficient management of resources, optimization of data flow, and enhancement of network security, contributing to the overall reliability and efficiency of WSNs. As these fields continue to evolve, they are poised to play an increasingly vital role in the advancement of WSN applications, offering new possibilities and improving the capabilities of these networks [17].

### 1.1. Semi-Supervised Clustering-Based Model

Another important scientific advancement, especially in the context of healthcare disease detection utilizing WSNs, is the semi-supervised clustering-based model.

1. Enhanced Disease Identification: This model incorporates semi-supervised learning methods into the clustering procedure, increasing the precision and dependability of disease identification based on signal processing. It improves the system’s diagnostic ability by enabling the detection of minute abnormalities or patterns in the gathered data.
2. Reduced False Positives: By combining labeled data with unsupervised learning, the model successfully lowers false positives in disease identification, making it a useful tool for healthcare practitioners who rely on accurate and useful insight.

### 1.2. Data Collection Using the DANA Algorithm

The dynamic adaptive node allocation (DANA) algorithm is a ground-breaking development in the area of wireless sensor networks (WSNs). Optimizing data collection procedures inside WSNs, with a focus on healthcare applications, are its main scientific breakthrough. Data are dynamically allocated and routed through sensor nodes in DANA to solve the issue of energy-efficient data collecting.

1. Dynamic Adaptation: DANA continually adjusts to the altering network parameters, such as node energy levels and data relevancy, to provide effective data routing while extending the network’s operational life.
2. Energy Conservation: By minimizing duplicate data transmission and dynamically assigning nodes to data collecting duties, DANA considerably lowers energy usage in WSNs, which is essential in healthcare settings with resource-constrained sensor nodes. This research seeks to illustrate the efficacy of the suggested technique in a healthcare use-case centered on signal processing-based disease identification. Traditional data collection techniques and unsupervised clustering algorithms are used to compare the performance of the DANA algorithm and semi-supervised clustering-based model.

### 1.3. Case Studies and Empirical Validation

The study uses case studies and empirical validations to show how well the semi-supervised clustering-based model and DANA algorithm work in actual healthcare settings. These studies offer verifiable proof of the usefulness of the scientific discoveries in real-world applications and their potential to transform healthcare through data-driven disease monitoring.

### 1.4. Problem Statement

The effective management and analysis of enormous datasets have emerged as key concerns across several sectors in an era marked by the exponential rise of data and the growing interconnection of systems. It is now more important than ever to have scalable and intelligent systems that can draw out valuable insights from different data sources. The challenge at hand in this situation is to create a cutting-edge data analysis framework that can manage huge and diverse datasets while simultaneously addressing the problems of interpretability, scalability, and flexibility. By enabling researchers, analysts, and decision-makers to extract useful information from complicated data structures, proposed method outperforms and should successfully be able to unleash the potential for innovations across a wide range of fields of study and real-world applications.

### 1.5. Motivation

The motivation behind this research is to develop an efficient data gathering approach for WSNs in healthcare settings, specifically focusing on signal processing-based disease detection. Traditional data gathering methods in WSNs, such as random or static schemes, are not optimized for healthcare use-cases, where real-time and accurate data are critical for timely diagnosis and treatment.

Utilizing the DANA algorithm, a reliable technique for data collection in wireless sensor networks (WSNs), together with a semi-supervised clustering-based model, offers a viable approach to problems unique to healthcare-focused WSNs. The data collection procedure is expedited by including the adaptive and dynamic routing capabilities of the DANA algorithm, as well as integration with a semi-supervised clustering model capable of effectively utilizing labeled and unlabeled data. The improvement of signal processing-based disease detection in WSNs is especially benefited by this optimization. This research specifically targets disease detection as a critical healthcare use case, emphasizing the importance of real-time and accurate data for timely diagnosis and treatment.

1. Conventional data gathering methods in WSNs, such as random or static schemes, are not well-suited for healthcare applications, where precision and timeliness are paramount.
2. By incorporating the DANA algorithm, renowned for its adaptive and dynamic routing capabilities, this research aims to harness its potential to enhance data gathering efficiency in healthcare WSNs.
3. Coupling the DANA algorithm with a semi-supervised clustering-based model allows for the efficient utilization of both labeled and unlabeled data, further optimizing the data gathering process for signal processing-based disease detection within WSNs.

### 1.6. Objective of Paper

1. To incorporate the dynamic adaptive node allocation (DANA) algorithm within WSNs and evaluate its performance in terms of data collection efficiency, energy conservation, and operational lifespan of the network.
2. To tailor the data gathering process to meet the unique demands of healthcare settings, ensuring real-time and accurate data acquisition which is crucial for prompt diagnosis and effective treatment.
3. To conduct a comprehensive comparison between the DANA algorithm, coupled with a semi-supervised clustering-based model, and traditional data collection methods as well as unsupervised clustering algorithms, establishing the superiority of the proposed method in terms of efficiency and accuracy.
4. To validate the effectiveness of the DANA algorithm and semi-supervised clustering-based model through practical case studies and empirical evidence, demonstrating their applicability and advantages in real-world healthcare scenarios.
5. To develop a state-of-the-art data analysis framework capable of handling large and diverse datasets, while simultaneously addressing challenges related to interpretability, scalability, and flexibility.
6. To empower researchers, analysts, and decision-makers within the healthcare sector, enabling them to extract valuable insights from complex data structures, ultimately contributing to the innovation and enhancement of disease monitoring and patient care.

### 1.7. Paper Organization

The remaining sections of this essay are structured as follows: The relevant research in the area of WSNs and disease detection is summarized in Section 2. The approach is described in Section 3, along with the DANA algorithm and semi-supervised clustering-based model. The experimental design and the analysis of the findings are presented in Section 4. The results and consequences of the suggested strategy are covered in Section 5. The work is concluded in last section along with recommendations for further research.

## 2. Related Work

The use of wireless sensor networks (WSNs) for healthcare applications has attracted a lot of study to improve data collection and disease diagnosis. To increase the operating life of resource-constrained sensor nodes, several researchers have looked into energy-efficient data aggregation methods designed for WSNs in healthcare. In order to increase accuracy and lower false positives, machine learning-based techniques for disease identification have been investigated [17]. It has also been studied the use of dynamic routing protocols in healthcare WSNs, emphasizing its importance in streamlining data gathering procedures. According to the goals of the proposed DANA algorithm and semi-supervised clustering-based model, these connected studies together contribute to the creation of effective and precise healthcare monitoring systems based on WSNs [18]. In the field of wireless sensor networks (WSNs) for healthcare applications, a wide range of related studies have been carried out to solve the urgent concerns of data collection effectiveness and disease detection accuracy [19]. In order to increase the operational lifespan of sensor nodes, which is essential in settings when resources are few, researchers have dug into the complexities of energy-efficient data aggregation approaches. Furthermore, supervised and unsupervised machine learning-based disease detection methods in WSNs have been investigated in order to improve diagnosis accuracy and reduce false positives. In order to optimize the gathering of healthcare data, the search for dynamic routing protocols in healthcare WSNs has also been a main focus.

Recent research has also explored the fusion of data from various sensor sources in WSNs, providing insights into how such data fusion can improve the accuracy and reliability of healthcare data, aligning well with the all-encompassing approach embodied in the proposed DANA algorithm and semi-supervised clustering-based model. Machine learning has been used to the field of real-time disease outbreak detection in order to quickly identify disease outbreaks using real-time sensor data, demonstrating the potential use of cutting-edge machine learning techniques in healthcare monitoring systems. Researchers have also looked on adaptive energy-efficient routing protocols, investigating ways to save energy while assuring data accuracy—a topic that is relevant to the DANA algorithm energy-efficient data collecting feature. Research on semi-supervised clustering methods is ongoing concurrently [20].

A thorough examination of the difficulties involved in integrating data from multiple sensor types and standards has also become a crucial concern for healthcare WSN interoperability concerns. This assessment emphasizes the value of a unified framework, a key concern addressed by the proposed DANA algorithm and integrated model, which seeks to harmony and expedite data collection procedures from diverse sources.

### 2.1. Signal Processing Techniques

There are frequently high spatial and temporal correlations between sensor data. Giving inadequate information in this situation is ineffective. The quantity of information supplied can be reduced by using the objective [21] and signal processing, particularly changes and encoding compression (CEC) approaches. The hub gathers data in accordance with the Shannon-SyQuest testing hypothesis to nearby CEC methods; these estimates are then changed and appropriately encoded. Additionally, the outcome of such a modification is sent away from the washbasin and stored in at least one packet in the payload. According to the particular application circumstances, specifically. Lossy algorithms are used to compress the raw data [22]. This enables the discarding of part of the original data. Increasing pressure levels while remaining beneficial with a certain degree of precision, the data may be replicated. Certain kinds of observation, however, need accuracy. It is implausible in some cases to expect that one will be aware of the scope of allowable observational errors without affecting the genuine facts. In addition, some application domains (such as body area networks (BANs)), where sensor hubs are continually present and filter and report critical information, call for sensors with high and cannot accept estimates that have been ruined by exactness. practices that include pressure loss. In this wide variety of WSNs, lossless data collection is both necessary and appealing. Authors of [23] have made suggestions for localized lossless compression plans. Lossy pressure strategies have been analyzed and investigated in relation to recreation mistakes and energy. Utilizations in [24], in this work, they emphasize lossless methods. They believed it to be out of place. Nevertheless, in some forms of verification, perceptual accuracy is crucial for comprehending the fundamental actual cycles. It is implausible to expect to be able to decide the amount of observational mistakes that are tolerable without compromising reliable data collecting in varied situations. As shown in Equation (1), some application areas (such as body region networks (BRNs), where sensor hubs continually monitor and record fundamental indicators) are required for sensors with high precision and cannot accept approximations polluted by lossy pressure processes.

The mathematical expression provided outlines a set of constraints concerning the 2-norms of vectors. In simpler terms, it suggests that when a vector x undergoes transformation through a linear operator or matrix, the 2-norm of the resulting vector is bound to stay within specific limits. More precisely, it will be confined to a range that is k times the original norm of x. This type of inequality is common in mathematical scenarios where maintaining the vector’s norm is crucial, such as in compressed sensing or in approximating matrices. The parameter k serves as a measure of allowance, ensuring the system’s fundamental characteristics are preserved, even with slight alterations, as represented in Equation (1).

(1) $$\left(1-\delta_{k}\right)∥x{∥}_{2}\leq ∥\Phi x{∥}_{2}\leq (1+\delta_{k})\|x\|$$

- $\delta_{k}$: This is the restricted isometry constant for sparsity level k. It quantifies how much the transformation Φ deviates from being an isometry (a distance-preserving operation) when applied to k-sparse vectors. A k-sparse vector is a vector that has at most k non-zero entries. The value of $\delta_{k}$ is between 0 and 1, and smaller values indicate that Φ better preserves the distances between sparse vectors.
- x: This is a vector in the original signal space. The vector x is what we want to recover in compressed sensing problems.
- $∥x{∥}_{2}$: This denotes the 2-norm (or Euclidean norm) of the vector x, calculated as the square root of the sum of the squares of its entries.
- Φ: This represents a linear operator or matrix that performs a transformation on the vector *x*. In the context of compressed sensing, Φ is often the measurement matrix that is used to acquire compressed measurements of the signal.
- Φx: This is the result of applying the transformation Φ to the vector x.
- $∥\Phi x{∥}_{2}$: This is the 2-norm of the transformed vector Φx.

### 2.2. Information Theory-Related Techniques

The focus of the data sink centric (DSC) clustering approach in wireless sensor networks (WSN) is on effective data collection and transmission to a single data sink. This method involves the formation of clusters by the sensor nodes on their own, with each cluster being run by a cluster head (CH) who is in charge of gathering and transmitting data locally. DSC reduces redundant transmissions by focusing on data aggregation at the cluster level, which saves energy and increases network longevity. The central sink is reached by the cluster heads after they have transmitted the aggregated data, enabling an organized and power-saving data routing process. DSC clustering is a good choice for applications that need real-time or near-real-time data, especially in cases like environmental monitoring when information is gathered from scattered sensor nodes across a wide region. These applications benefit from dynamic adaptability and decreased latency.

The association of data concurrently secured by many sensors may be employed by DSC approaches, which are driven by the Slepian–Wolf assumption [25]. The DSC systems state that each sensor center point transmits its compressed results to the sink for collective interpretation. This indicates that during pair gathers, the center points must cooperate for one to contribute side information and the other to compress it to the Slepian–Wolf or Wyner–Ziv limit. Due to their foundational belief that the full benefits of the secret data appointment should be understood early on [26], DSC techniques are similarly challenging to implement in these situations. The most popular and useful DSC implementation is called DISCUS [27], where sensor center points are thought of as being divided into bundles. As side information, a center point (the collecting head) communicates uncompressed data for each pack, while any additional centers send encoded (i.e., compacted) data. Permit us to assume that (quantized) assessments will fall between [0, 7] for the whole number and that, when taken into consideration, all data from different sensors will be discriminated at (almost) the same time contrast. To address unambiguous data from each sensor with regard to wireless sensor networks (WSNs), the DANA algorithm, or the semi-supervised clustering-based model, “DSC” is not a widely used acronym or abbreviation. It is possible that “DSC” refers to a specific term or concept within a particular research or project context. It is difficult to give a detailed answer without more background or details. A WSN generally comprises of several small sensor nodes with limited resources that are dispersed across a vast region. These nodes have sensors to gather data, computing units to execute calculations, and wireless communication capabilities to send data to a centralized base.

In this case, sensor nodes in a wireless sensor network (WSN) are grouped into clusters, with each cluster having a designated cluster head in charge of data aggregation. In order to communicate with the cluster head, the sensor nodes encode the features they have seen. In particular, the binary representations 00, 01, 10, and 11 are used to express independently properties that are thought to be similar and have a base distance of 4. The cluster head then groups these encoded traits into four unique bins, numb0, 4, 1, 5, 2, and 3, and stores them in a common repository. When a sensor node sends data with the encoded value 01, the sink, the main data gathering point, instantly decodes it as 5, identifying the value as 5.

### 2.3. Clustering-Based Big Data Gathering in Densely Distributed WSN

When examining the arrangement of data gathering in WSN using a flexible sink, the best strategy to decrease energy usage is to pick where data collection is coordinated. At the end of the day, a response to the process with two inquiries will not be more significant than this issue. (1) Which computation is the most effective for classifying centers into packs? (2) To decrease energy usage, how many clusters are advised? Reduce the square of data transmission distance inside an organization using the best bundling calculation to lower energy consumption for data transmission. This is because, according to our predictions, the energy used for data transmission at the center is inversely correlated with the square of the transmission distance [28]. Similarly, according to the statement, there is an inverse square relationship between the transmission distance square and the amount of energy used to transmit data at the center. This connection is frequently linked to the free-space route loss model in wireless communication, which describes how signal strength declines as transmitter and receiver distances rise. The received signal power (received P received) is inversely proportional to the square of the distance (d) between the transmitter and receiver in free-space route loss. This connection is denoted mathematically by the following. The formula below shows how energy usage for data transmission is frequently correlated with transmitted power (transmitted P sent).

**Figure 1:**
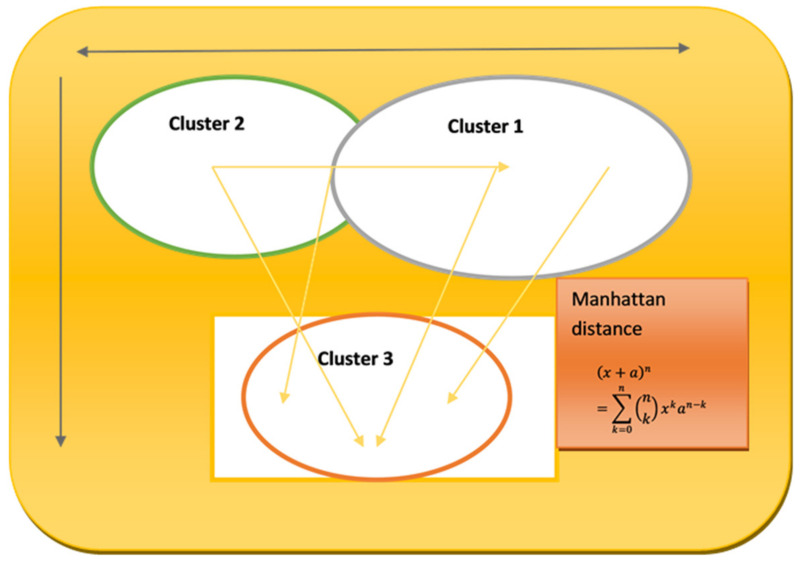
Clustering networking model.

Its adaptability is demonstrated by the model technique’s use in a variety of dynamical systems, including rare occurrences and high-degree-of-freedom systems like boundary layer flow simulations, classic issues like the Lorenz attractor, and other dynamical systems. This strategy works well even for complicated problems with a lot of degrees of freedom, providing computational solutions without assuming any particular analytical structure for the model. In Figure 1, the model expands its value when used in conjunction with a clustering networking model, where clusters are modeled using various distance measurements and algorithms, such as Manhattan distance. The aforementioned model is utilized in a situation where N sensor centers, each one representing a different circle, are present. To complete the assignment, an adjustable washbasin must go through K focus points of bundles, each of which is addressed by a filled circle [29]. This implies that the model method is flexible enough to handle the challenges of clustering in networking, supporting a variety of distance measurements and algorithms to efficiently move between and control the sensor hubs in the network.

Equation (1) represents the binomial theorem, expressing the expansion of the $power{\left(x+a\right)}^{n}$. Here, x and an are terms being raised to the power of n. The symbol ∑ denotes a summation, k is the index of summation, and (n!k) are binomial coefficients, which determine the number of ways to choose k terms from n, aiding in the distribution of the powers across the terms. The terms $x^{k}\;and\;a^{\left(n-k\right)}$ represent the varying powers of x and a in each term of the expansion. This theorem is fundamental in algebra for expanding expressions that are raised to a power.

Table 1 lists some strategies for acquiring data in wireless sensor networks (WSNs) that is energy-efficient across a variety of properties. Significant factors include heterogeneity, mobility, space complexity, memory utilization, length of input data, clustering goals, and big data aspects like volume, variety, and efficiency. The suggested approach stands out by addressing these characteristics thoroughly and including elements like overhead reduction, low-latency interference reduction, storage optimization, energy-efficient data collection, and sophisticated analytics. By maintaining efficiency in resource utilization and performance across several WSN variables, this approach tries to achieve a balance.

### 2.4. Research Gap Findings and Contribution

As Shown in Table 1, the use of cutting-edge algorithms and intelligent sensor technologies results in the proposal of a revolutionary, energy-efficient system model. The approach reduces energy consumption and maximizes resource utilization in wireless sensor networks (WSNs). The system effectively adjusts to shifting workload conditions by utilizing a dynamic power management strategy and sophisticated data aggregation algorithms. As a result, the sensor nodes’ lifespan is greatly increased while ensuring optimal performance. Robust simulations and empirical investigations are used to demonstrate the model’s performance and highlight its potential for deployments of sustainable and long-lasting sensor networks. A novel method for data collection in WSNsdesigned for signal processing in disease diagnosis in the healthcare industry is the DANA algorithm in conjunction with a semi-supervised clustering-based model. By utilizingneighborhood analysis, the DANA algorithm optimizes the data aggregation process and enables sensors to effectively participate in the collection and aggregation of pertinent data. By including labeled and unlabeled examples, the semi-supervised clustering-based model significantly refines the collected data. By using both labeled training data and unlabeled real-time sensor data, our hybrid model improves the accuracy of diseasediagnosis. The model adapts to the dynamic nature of healthcare-related data by iteratively improving its clustering and classification procedures [30].

Although legitimate, the worry over “real-time” in the context of data latency is essential for healthcare applications. Determining the specifics of real-time data processing is crucial for the essay. In order to satisfy the demands of particular applications, real-time processing often includes minimizing data processing and transmission delays. Within the task to determine the system’s capacity to give timely insights, the delay in data transmission and processing must be clearly established. In the healthcare use case, take into account clearly defining the maximum tolerable latency for data processing and transmission. For instance, if the application requires almost immediate answers for crucial patient monitoring, specify the acceptable delay in terms of milliseconds or seconds. By addressing this issue, the suggested model is guaranteed to comply with the demanding real-time standards of the healthcare industry.

An optimization issue that aims to reduce a system’s energy consumption (***E***) while satisfying certain performance requirements (***P***) is known mathematically as an energy-efficient system model. This may be expressed as E=f(P), subject to restrictions on power distribution ($P_{i}),$ component efficiency (I), and operating parameters (Xian), among other factors. It may be stated mathematically as: In equation given consideration of the restrictions $P_{i}P_{max},X_{i}X_{max}$, and any pertinent restrictions, minimize ESHA $=(P_{i}*t_{i}*X_{i})$ for all components i. By modifying power allocations and operating parameters within predetermined bounds to maintain acceptable performance levels, the goal in this situation is to reduce total energy usage. In a variety of disciplines, this mathematical framework enables the systematic design and optimization of energy-efficient systems.

## 3. Material and Methods

The DANA algorithm is responsible for optimizing data gathering and routing within the WSN. Routing decisions (A(i,j,t)) It appears that A(i,j,t) represents an element in a matrix or an array given. The third dimension—time or another variable—could be represented, respectively, by the indices I, j, and t. The element in the it row, j-the column, and t-the layer (or time step) in a three-dimensional matrix A, for instance, would have the value A(i,j,t). The exact routing algorithm and associated equations will depend on the specifics of the DANA algorithm used. It may involve considerations such as the distance between nodes, node energy levels, and the relevance of data. In order to improve data collection and routing in WSNs, the DANA (distributed adaptive network analysis) algorithm is applied. It seeks to conserve energy while effectively gathering and transferring data to a base station. Although the DANA method may be implemented in a number of different ways, there is not a single accepted mathematical representation for it.

As part of the pre-processing phase of signal analysis, it is usual practice to segment samples into brief frames in order to guarantee that the signals are quasi-stationary, meaning that their statistical features are roughly consistent within each frame. Analyzing the signal’s properties over time is made simpler via segmentation. There are 30 milliseconds between each frame in this scenario. A 60% overlap is used, which means each frame shares 60% of its content with the one next to it, in order to prevent jarring transitions between them. The signal’s continuity is preserved by this overlap. After that, the Hamming window function is used to window. Each frame’s edges are subjected to a mathematical function called the Hamming window, which smoothes and taper the edges. Each data point in the frame will be multiplied [31].

As we have discussed in the previous section, we can think of the specification of the kernel matrix as a way to express prior knowledge. Consider a given similarity function of the form K :X×X→R. Is it a valid kernel function? That is, does it represent an inner product between $\psi (x)\;and\;\psi (x_{0})$ for some feature mapping. The following lemma gives a sufficient and necessary condition. A symmetric function K:X×X→R implements an inner product in some space if and only if it is positive semi definite; namely, for all $x_{1},\;\ldots ,\;x_{m}$, the Gram matrix $G_{i,j}=K(x_{i},\;x_{j}),$ is a positive semi definite matrix [32].

A clustering technique is the foundation of a first naïve dictionary learning method let us say we learn the function c: $X_{1},...,\;k,$ where c(x) represents the cluster to which x belongs. Then, we may conceive of the clusters as “words” and the instances as “documents,” where a document x is mapped to the vector (x) 0, 1 k, where (x) I is 1 if and only if x belongs to the it cluster. It is now clear to understand that using a linear predictor on x is similar to giving all instances that are part of the same cluster the same goal value. Additionally, if the grouping is based on distances from a class centre (such as k-means), the auto-encoders methodology, which is more broadly applicable to dictionary learning, may be seen as a particular instance of both the k-means and PCA methods. In an auto-encoder, we learn two functions: an “encoder” function, $R_{d},\;R_{k}$, and a “decoder” function, :$R_{d},\;R_{k}$. Finding a pair of functions whose reconstruction error, $P_{i}k_{xi}(x_{i})$ is minimal is the aim of the learning process. Of course, we can simply set k = d and both, to be the identity mapping, producing a flawless reconstruction. Therefore, we need to limit both. In PCA, we further limit k and d to linear functions. In k-means, k is not constrained. Equation (2)equals a cost function called 2 and is frequently employed in optimization issues, notably in simulations of the dynamics of interacting entities. Theoretical considerations will now be discussed. The cost connected to the expected future locations of entity k and its neighbors is represented by (,) Joke (*v*(*k*, *i*)) in the context of dynamic systems. The expression in k sums over all nearby entities aside from k itself.

(2) $$J_{k}(v\left(k,i\right))=\sum l\in N_{k}\left\{k\right\}{\left[∥\left(x_{k,i}+∆tvk,i\right)-\left({x_{l}}_{i}+∆t\;v\_(l,i\right)-r\right]}^{2}$$

- $J_{k}(v(k,i))$: This is a function that seems to calculate a cost, energy, or discrepancy associated with the velocity v(k,i) of the k-th agent or particle at time instant i.
- $\sum_{l\in N_{k}}$: This represents a summation over all l in the neighborhood $N_{k}$ of the k-th agent. $N_{k}$ could be a set of indices representing the neighbors of the k-th agent.
- $x_{k,i}$: This represents the position of the k-th agent or particle at time instant i.
- $v_{k,i}$: This represents the velocity of the k-th agent or particle at time instant i.
- Δt: This is a time step or increment. It represents the difference in time between two consecutive instants.
- r: This seems to be a constant vector, possibly representing a desired relative position or offset between agents in the system.

In Equation (2), controls the pursuit of a target under a positive scaling factor restriction in the setting of WSN. However, depending on (4) implies knowing the aim beforehand, which is frequently unfeasible. To solve this problem, we substitute the unavailable w o with local estimates, *w*(*k*, *i*) (explained later). A certain distance r from neighbors is maintained by the motion decisions of nodes by taking collision avoidance into account. The first component includes the neighborhood average velocity for node k. The second term, which is a linear combination of the displacement vectors $x_{l},\;i\;and\;x,k,\;i,$ recommends that nodes should alter their velocities to match the neighborhood’s typical displacement vector while maintaining an r-distance between them [33]. In this context, the objective is to estimate the target’s location relative to an origin coordinate using data and observations from multiple nodes in a distributed wireless sensor network (WSN). Each node knows its location within a global coordinate system. The distance between the target and a node is represented by an inner product involving unit direction vectors and measurement noise. A linear regression model is derived from this relationship. To estimate the target’s position in a distributed manner, nodes minimize a cost function, which they cannot optimize individually. Instead, they employ the ATC diffusion algorithm to adapt and combine information from neighboring nodes. Additionally, the nodes aim to estimate the velocity of the center of gravity, “vg”, in a distributed manner. A global cost function is introduced to facilitate this estimation over time. The diffusion algorithm for “vg” estimation involves intermediate variables and coefficients that satisfy certain properties. Overall, this approach enables nodes in the WSN to collaboratively estimate the target’s position and velocity in a distributed and energy-efficient manner.

### 3.1. Different Data Collection Techniques Using Clustering

In WSNs, energy-efficient controlling is carried out via different evened-out or bundle-based coordination. Different gathering estimations have been developed and improved in a study to prolong the life of sensors in WSNs while they are doing their responsibilities. Sensors are divided up into regional bundles with a reasonable number of groups. Each bundle selects a part pioneer, known as the gathering head (CH), who will be responsible for moving all gathered data from the bundle to the sink or the business server (BS). The sensors on non-bunch heads only transmit their data on their own Cheshire are several approaches to gathering calculations. While still more emphasize the necessity of selecting energy-efficient topography for the association, others concentrate on the number of gatherings in WSNs. Others focus on the separations between CHs and BS or between CHs and sensors that are not CHs. All strategies for evened-out data variation have the following universal components in common.

K-mean clustering: In WSNs, K-suggests is a very basic yet efficient approach [32,33]. We anticipate having many sensor center points, $X=[x_{1}x_{2}...\;x_{N}]$, and we need to organize them into Nc packs, each of which contains a bundle head (CH) at the center. There are just the following four necessary steps in the computation.

I. For NC gatherings, choose centric centers at random (or based on historical data); initially, this truly has no bearing on the selection of these conditions. Calculate the cross-section of the gathering model $M=[m_{1}m_{2}...\;mNc]$.
II. For example, choose the closest group Cow to which you should assign each item in the educational record.
  (3) $$x_{j}\in Cwif\left|t\right|x_{j}-mw||<\left|x\right|x_{j}-m_{i}||$$
  - $x_{j}$: This represents an element from a set, which could be a vector or a value depending on the context.
  - $C_{w_{i}}$: This seems to represent a subset or a class of elements, indexed or parameterized by $w_{i}$.
  - t: This is a scalar that might control the scaling of the distance between $x_{j}$ and $m_{w}$. It could be a threshold or a parameter of the system.
  - $m_{w}$: This is likely a centroid, mean, or reference point in the space of $x_{j}$, associated with the subset $C_{w_{i}}$.
  - $∥x_{j}-m_{w}∥$: This represents the distance (possibly Euclidean distance if $x_{j}$ and $m_{w}$ are vectors) between $x_{j}$ and $m_{w}$.
  - x: This is another scalar, possibly a weight or another parameter influencing the decision boundary for classifying $x_{j}$.
  - $m_{i}$: This is likely another centroid, mean, or reference point in the space of $x_{j}$.
  - $∥x_{j}-m_{i}∥:$ This represents the distance between $x_{j}$ and $m_{i}$.
III. The spacing between a CH and non-CH sensor is taken into account when we improve bunches at this step. A sensor will select the nearest CH to be with, and new CHs should be close to the point of pack convergence [34].
IV. Calculate the gathering model’s cross-section once again while considering the continuous distribution.

**Figure 2:**
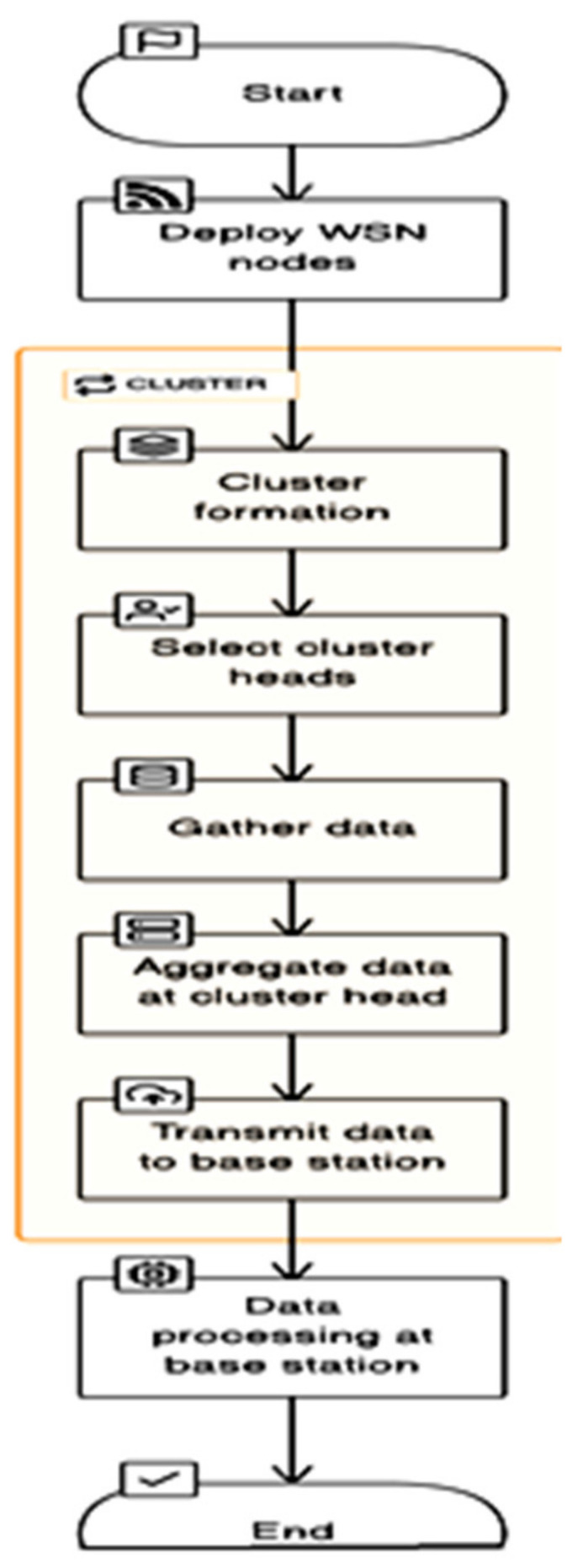
Proposed k-mean + DANA clustering approach.

The suggested clustering method is shown in Figure 2 and consists of a cosine similarity function for threshold optimization together with K-means clustering and the DANA technique. Data are divided into k groups based on similarity using the well-known clustering method known as the K-means algorithm. K-means is probably used for the first grouping of data points in the suggested strategy. For effective data collection in WSN. For data transmission to be optimized, energy consumption to be decreased, and network efficiency to be increased, it frequently includes cooperation among sensor nodes. In order to identify how similar two data points are in a multi-dimensional space, the cosine similarity function $\left(s_{ij}\right)$, a measure of similarity between two vectors, is frequently employed in clustering. By guaranteeing that data points are correctly categorised according to their cosine similarity, it probably contributes to this situation’s optimisation of clustering thresholds. In order to achieve a more effective and energy-efficient grouping of data in a wireless sensor network, Figure 2 shows the integration of K-means and DANA in a clustering technique. Thresholds are optimised using the cosine similarity function.

The FCM (fuzzy C-means) clustering method surpasses K-means owing to its improved speed and more efficient energy allocation, according to current suggestions [35], which suggest the use of batch estimate. FCM, as opposed to K-means, permits a sensor to be a member of many cluster heads (CH), taking into account their connections or degrees. In a situation where N sensor nodes are dispersed throughout c clusters, the method seeks to reduce the overall energy within clusters, represented as Jm. The it cluster is connected to the degree of the center j, denoted by the symbol unit. The symbol Dig denotes the separation between the clusters’center and the center j. For our WSNs, the model cross-section or cluster center is indicated as M node x [35].

### 3.2. LOW-Energy-Abled Hierarchical Clustering (LEACH)

**Figure 3:**
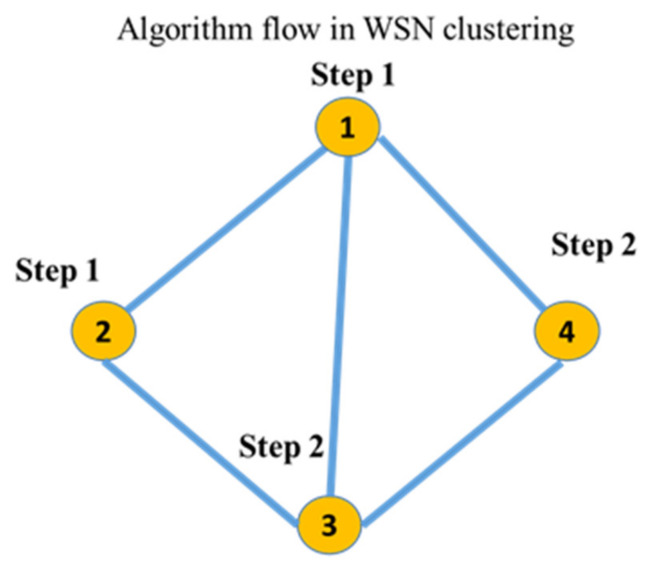
LEACH algorithm.

In the setup phase of the LEACH algorithm, nodes initialize and choose cluster heads (CHs) at random. Data is sensed by nodes, transmitted to CHs, aggregated, and finally transmitted to the base station during the steady-state phase. During this phase, data is sent (from non-cluster nodes to CHs) and CHs rotate on a regular basis. The method assures energy-efficient data collection by grouping nodes into clusters that enable nodes to transfer data to neighboring CHs while using less energy. This flowchart summarizes the key processes of LEACH, which optimizes energy consumption in WSN in Figure 3 [36].

### 3.3. Data Collection Algorithm for Random Walks Based on Compressive Sensing (CSR)

We model a WSN with N reliable sensors that are randomly distributed in either a square with a district of L or a circular region with R0. An erratic numerical chart G depicts the geology of the linked organization (V, E). Each sensor is expected to have a telecom length (also known as transmission) that is comparable to a Euclidean distance. It is also expected that sensors would be able to modify the amount of energy they require to send data to take into account their real distances from one another. We cannot compare our association to a conventional association since each sensor has a unique number of neighbors within the range R. The graph’s link will change as follows if we change the value of R: Vertex V’s strategy compares when all sensors are considered, but edge set E changes when the relationships between sensors in each region are taken into consideration. We predict that to organize the connection, each center point of interaction with O(logN) neighbors will be selected at a genuine R-value [36].

### 3.4. ECG (Electrocardiogram) Theory

**Figure 4:**
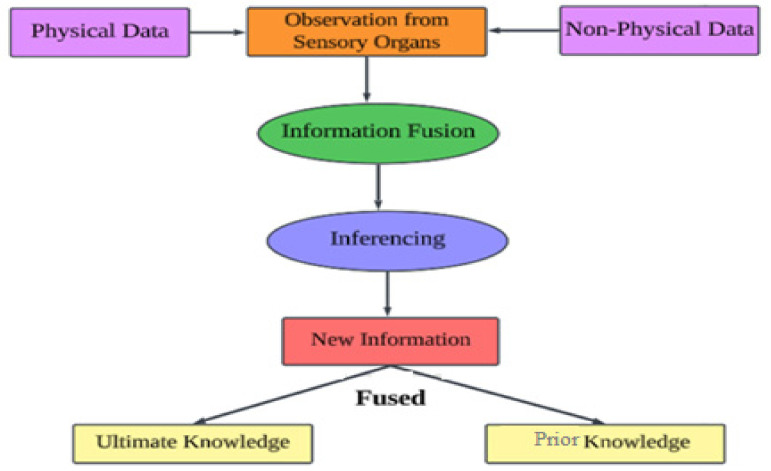
Proposed method.

**Figure 5:**
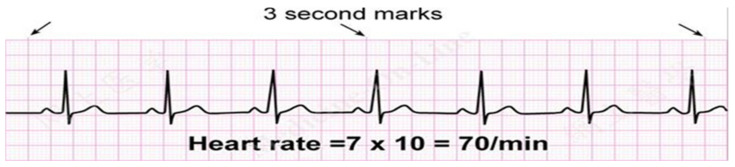
Example of Normal ECG Graph recording.

An ECG is a healthcare tool used to record a person’s heart’s health. ECGs function by receiving cardiac electrical signal abstractions and displaying them in a PQRST graph, a type of visual anatomy. Electrode placement at various places allows for the acquisition of the heart’s electrical signal. To receive the electrical signal from the heart, 12 leads of electrodes are typically employed. By observing the PQRST wave’s structure, amplitudes, and wave periods, the condition of the heart may be determined. Figure 4 and Figure 5 displays a typical ECG graph, with the time constant (s) indicated by the horizontal axis and the amplitude of the graph (mV) by the vertical axis. The ECG graph is recorded on a moving piece of paper following the cardiac activity tracked by the electrodes.

Then, the recorded wave pattern and normal wave pattern from every 12 leads are set side by side by the physician. Any irregular pattern may approve of certain abnormalities or an unhealthy state of the heart. But the information assembled from this graph is not sufficient to conclude heart block or an arrhythmia, which is why the theoretical data of heart conditions and how to respond to that ECG graph is collected to draft a conclusion about the graph obtained. Those data are listed in Table 2. The provided table includes many electrocardiogram (ECG) hypotheses together with the accompanying findings, acting as a thorough diagnostic manual for heart diseases. Notably, positive R waves in certain leads are indicative of normal baseline and standard norms, whereas variations in the direction of R waves can reveal changes in the left and right heart’s orientation. A typical heartbeat presentation indicates normal cardiac rhythm, but anomalies such bundle branch blockages (RBBB, LBBB), cardiac arrhythmias, atrial tachycardia, and ventricular tachycardia are recognized by different ECG patterns. The table gives medical professionals a quick yet comprehensive reference for understanding ECG data, assisting in the quick, yet precise diagnosis of a variety of heart disorders based on particular electrocardiographic symptoms.

### 3.5. Proposed Method

The DANA algorithm employs a decentralized approach, which is in line with the characteristics of WSNs and was inspired by the idea of distributed antenna systems. The semi-supervised clustering approach makes the best use of both labeled and unlabeled data in the network, which is an important tactic in healthcare settings where acquiring labeled data for various health problems may be difficult. Data collection for real-time monitoring within the WSN is the main goal. Sensor readings pertaining to health metrics are collected. Signal processing methods like filtering and feature extraction are used to the obtained data in order to boost pertinent information and speed up disease detection. Its potential for remote patient monitoring and prompt intervention is highlighted by the use of this approach in healthcare. Because there is a lack of labeled data in the healthcare industry, the approach’s semi-supervised nature tackles this issue. In addition, the semi-supervised clustering model’s integration adds flexibility to respond to shifting network circumstances and dynamic healthcare settings. The model takes into account the variety of sensor data, allowing for differences in data patterns linked to various disorders. The algorithmic combination attempts to reduce energy consumption, a crucial component in WSNs, by strategically directing antennas and optimizing data collection paths. Scalability and robustness are guaranteed by the antenna network’s distributed design, which is essential for large-scale healthcare installations. The suggested approach also places a strong emphasis on real-time processing, which makes it possible to quickly identify abnormalities or disease early warning indications. Concerns about delays in data transmission and processing are addressed by the algorithmic framework, which provides parameters for real-time limitations. Since prompt identification may have a big influence on patient outcomes in healthcare applications, this becomes especially important. The conversation arises when it is not clear whether expecting the benefits of screening offsets the risks of the screening approach itself and any resulting illustrative tests and medications. Screening tests ought to find success, secured by and large, by perseverance with acceptably low speeds of sham positives and deceiving unfriendly results. If signs of sickness are distinguished, more convincing tests are performed to support the finding. Assessment for harmful development can result in sickness avoidance and an early end. Early assurance could result in faster treatment and longer life. In any case, it could also wrongly appear to extend the hour of death through lead time tendency or length time inclination [37].

#### Knowledge Graph System KGS Theory

In a graph-like structure, a knowledge graph system (KGS) represents knowledge by encoding entities, their properties, and the connections between them. The main parts of a knowledge graph are nodes, which stand in for entities, edges, which represent links, and properties, which reflect qualities of either entities or relationships. The principal parts are described below. A knowledge graph system (KGS) is a complete framework for classifying and displaying data that embodies things as nodes and their connections as edges in a graph structure. Each node represents a different entity, such as a person, concept, or item, while edges show the relationships and affiliations between these things. Nodes and edges are enhanced with extra information by properties, such as traits or characteristics. KGS enables complex queries and conclusions by facilitating the effective retrieval of related knowledge [38]. KGS is a strong tool for modelling complex relationships within data, promoting a nuanced knowledge of information networks. It is widely used in a variety of fields, such as artificial intelligence and semantic web applications.

The electronic structure of atoms and molecules is described by the KGS hypothesis, a key idea in quantum mechanics’ density functional theory (DFT). The KGS theory may be described mathematically using the examples below. In DFT, a system’s overall electronic energy is represented as a function of electron density of nodes:

(4) $$E\left[\rho \left(r\right)\right]=T\left[\rho \left(r\right)\right]+Vext\left[\rho \left(r\right)\right]+E_{Hartree\left[\rho \left(r\right)\right]}+E_{xc\left[\rho \left(r\right)\right]}\;\left[\rho \left(r\right)\right]\approx -\int 1\rho \left(r\right)V_{KS\left(r\right)dr}$$

Equation (4) represents the total energy E of a quantum system in terms of electron density ρ(r).T is kinetic energy, $V_{\mathrm{ext}\;}$ is external potential, $E_{\mathrm{Hartree}}$ represents electronCoulomb interactions, and $E_{xc}$ accounts for exchange-correlation effects. In this case, E stands for the total electronic energy, T for the electrons’ kinetic energy, Vext for the external potential, which is often caused by atomic nuclei, $E_{Hartree}$ for the Hartree energy (the electrostatic energy resulting from the attraction of electrons to one another), and $E_{xc}$ for the ex-change-correlation energy. The kinetic energy term T[(r)], which is a key component of the KGS theory. It suggests that a set of non-interacting electrons in an effective potential, known as the Kohn–Sham potential $(V_{KS}\left(r\right)),$ may be used to approximation the kinetic energy of the electrons in a multi-electron system:

Equation (4) approximates the kinetic energy functional T[ρ(r)] of an electron system. It integrates the product of electron density ρ(r) and Kohn–Sham potential $V_{KS}(r)$ across space. The kinetic energy of electrons that are not interacting in the Kohn–Sham potential is set up so that it roughly resembles the actual kinetic energy of electrons that are interacting. The Kohn–Sham equations are described by the following formulas:

(5) $$\left(-½\nabla^{2}+V_{KS\left(r\right)}\right)\varphi_{i\left(r\right)}=\varepsilon_{i}\varphi_{i\left(r\right)}$$

This Equation (5) represents the Kohn–Sham equation in density functional theory. Here, $-\frac{1}{2}\nabla^{2}$ is the kinetic energy operator, $V_{KS}(r)$ is the Kohn–Sham potential, $\phi_{i}(r)$ are Kohn–Sham orbitals, and $\epsilon_{i}$ are their corresponding eigenenergies.

Here, $e_{i}$ stands for the appropriate eigenvalues (energies) of the Kohn–Sham orbital’s (one for each electron), and $V_{KS}\left(r\right)$ is the Kohn–Sham potential. The Kohn–Sham potential is determined from the square of the Kohn–Sham orbitals, which is the electron density for the system:

(6) $$\rho \left(r\right)=\sum >{\left|\varphi_{i\left(r\right)}\right|}^{2}$$

The Kohn–Sham potential is then determined by a self-consistency procedure, typically iteratively adjusting V_KS(r) until the calculated electron density ρ(r) matches the actual electron density of the system.

From Figure 4, combining data from several sensors to derive inferences or make predictions is the process of fusion and inference of sensory data.

The algorithmic difficulty, input data volume, data fusion strategies, inference model selection, and processing requirements all have a role in how complicated the suggested model is. The time and spatial complexity of the model might be drastically different depending on these variables. In addition, factors like hardware acceleration, scalability, parallelization, and model maintenance add to the complexity overall. To determine if using the model in real-world situations is feasible, especially in circumstances with limited resources or a tight timeline, a thorough analysis of these components is necessary [38].

### 3.6. KGS for Inferring ECG Data of Arrhythmia and Heart Blockage

In this paper, perceptions are restricted to knowing about the inclination of heart block and arrhythmia. To recognize blocks in the group branch, the estimation of QRS point can be utilized [39,40]. In any case, the strange state of heartbeat mood is arrhythmia. Those two conditions can be utilized for any coronary disease as pre-recognition data. To carry out KGS calculation, first, gather speculation data and signs identified with the perception of ECG came about because of heart states of arrhythmia heart blockage. Here, eight conditions of the heart are attracted and two situations are speculation data. Figure 5 shows the eight theories and signs identified with ECG perception results [41]. These outcomes will become data on heart conditions. In CAI programming, hypotheses and indications can be found in Table 3.

### 3.7. Proposed Model for Data Gathering Technique

**Figure 6:**
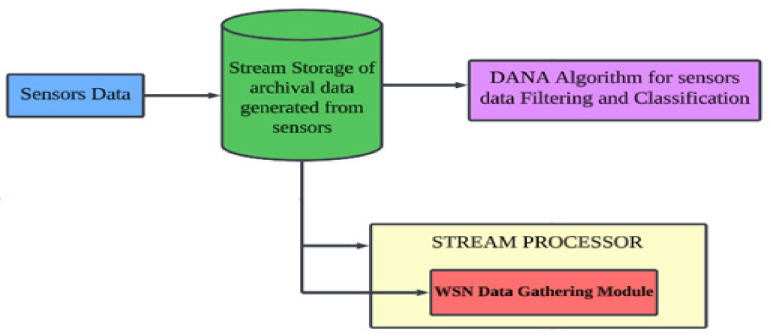
Data Steam Management.

A stream processor may be compared to an informational index organization system in terms of data the board structure, the important level relationship of which is suggested in Figure 6. The building may receive several streams. The duration between portions of one stream does not have to be uniform, and each stream is free to supply pieces according to its schedule. They also do not need to have comparable data rates or data types. The final structure manages the speed at which data is examined from the plate, so it never has to be concerned about data loss while attempting to execute queries. Although streams may be noted in a massive archive, we anticipate that taking notice of requests from the archive will be implausible. Functional storage is also included, which may be used to store requests as well as summaries or pieces of streams. The working store can be either a plate or a crucial piece of memory, depending on how rapidly we need to respond to requests. It is unquestionably constrained enough to prevent it from being able to retain all of the data from each stream, though. Let us take a moment to consider how frequently stream data occurs before going on [42].

A situation where a sensor, originally measuring the ocean’s surface temperature, develops into a more complex instrument with the inclusion of a GPS unit in the world of healthcare and the Internet of Medical Things (IoMT).

Let us consider the use of a data stream amendment approach in relation to Figure 6, which depicts a data stream storage system. In order to distribute the final m centers precisely throughout k groups while allowing for flexibility in the number of groups, this method uses models to steer the merging of smaller bundles into larger ones. Geographical placement of stream points is unlimited for inquiries about centroids in Euclidean or non-Euclidean domains. The task becomes simpler if we realize that each stream segment is selected using data that is consistent across the stream. The streaming paradigm in this scenario assumes that the stream elements’ values may change over time, leading to dynamic behaviors including expansion, contraction, splitting, merging of bundles, or continuous movement.

## 4. Simulation and Results

Table 4 presents an example of time-series data clustering using several clustering methods (Fuzzy, Leach, K-Mean, DANA) and distance metrics. The columns reflect the membership values supplied by the algorithms and the rows show various time points (T1 to T5) and clusters (K1 to K8), respectively. The entries indicate the strength of the relationship or membership that data points have with specific clusters at a given moment, with 1 being complete membership and 0 denoting none. The use of several distance measurements, ranging from 1 to 5, particularly emphasizes how the algorithms take into account diverse metrics. The table gives a thorough overview of how different algorithms distribute data points across clusters over a range of time points, illustrative of the dynamic character of the clustering procedure [43].

In this table, the correlation hypothesis across different algorithms is analyzed over time (T1–T5) and across various clusters (K1–K8). The ‘Distance Measures’ columns (1–4) represent the distances between the nodes within each cluster. ‘Fuzzy’,‘Leach’,‘K-Mean’, and ‘DANA’ columns represent the correlation values or probabilities associated with each cluster for the respective algorithms. A value of 1 indicates a strong correlation or high probability of the cluster being selected, while a value of 0 indicates no correlation.

In Table 1, a comparative study of state-of-the-art methods is presented, analyzing their performance using different methodologies.

Effectively measure and evaluate the proposed work, which involves the implementation of the dynamic adaptive node allocation (DANA) algorithm within wireless sensor networks (WSNs) for healthcare applications, several key metrics will be utilized. These metrics are crucial in assessing the performance, efficiency, and overall impact of the proposed system. Here is a list of the metrics that will be used:

1. Network Lifetime: Measure the operational duration of the WSN from the start until the first sensor node runs out of energy. The longer the network lifetime, the more efficient the data gathering process is in terms of energy conservation.
2. Energy Consumption: Assess the average energy consumed by the sensor nodes during the data gathering process. A lower average energy consumption indicates a more energy-efficient system.
3. Data Accuracy and Reliability: Evaluate the accuracy of the data gathered by the WSN, ensuring that it is dependable and precise, which is crucial for healthcare applications.
4. Latency: Measure the time taken for data to travel from the sensor nodes to the base station. Lower latency is essential for real-time applications like healthcare monitoring.
5. Data Redundancy: Assess the level of redundant data transmitted in the network. The goal is to minimize data redundancy to conserve energy without compromising the quality of data collected.

**Figure 7:**
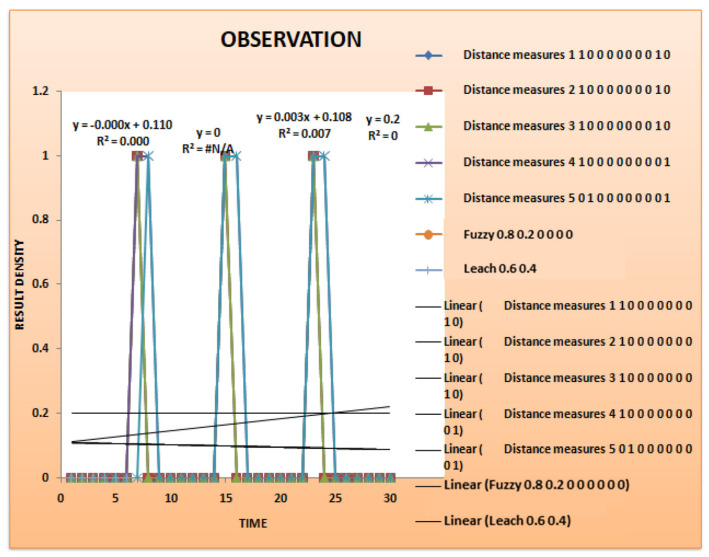
Example Observation Result Graph time by time.

A representation of the results of clustering over time is shown in Figure 7, the example observation result graph. The *y*-axis shows the degree of membership or relationship between individual data points and certain clusters, while the *x*-axis shows different time points (from T1 to T5). A different cluster is represented by a coloured line, and the trajectory of each coloured line across the time axis shows how the membership values evolve over the observation period. Stronger associations are shown by peaks in the lines, whereas transitions or reassignments may be represented by dips. Insights into the temporal patterns and trends that the clustering algorithms have been able to capture may be gained by analysing this graph, which demonstrates dynamically how data points change in their cluster membership across successive time intervals [44].

**Figure 8:**
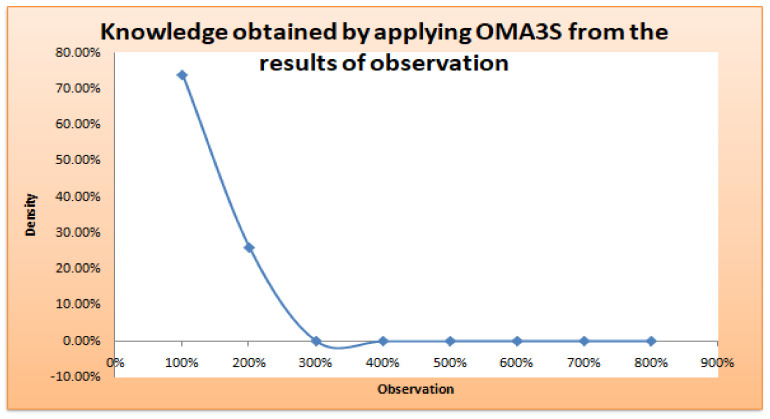
Information acquired by applying OMA3S from the consequences of perception.

The insights, patterns, or knowledge discovered via the process of analyzing and interpreting observational data using the OMA3S (object-oriented moving average space-based surveillance) system are referred to as the information collected by applying OMA3S from the results of observation. OMA3S is probably a monitoring or surveillance system that uses an object-oriented moving average method for geographical analysis. The information gathered may consist of geographical trends, item motions, irregularities, or any other pertinent patterns found in the observed data. It suggests that OMA3S is used to draw out important knowledge or information from the observations, aiding in a better comprehension of the processes or things that are being seen in Figure 8.

**Figure 9:**
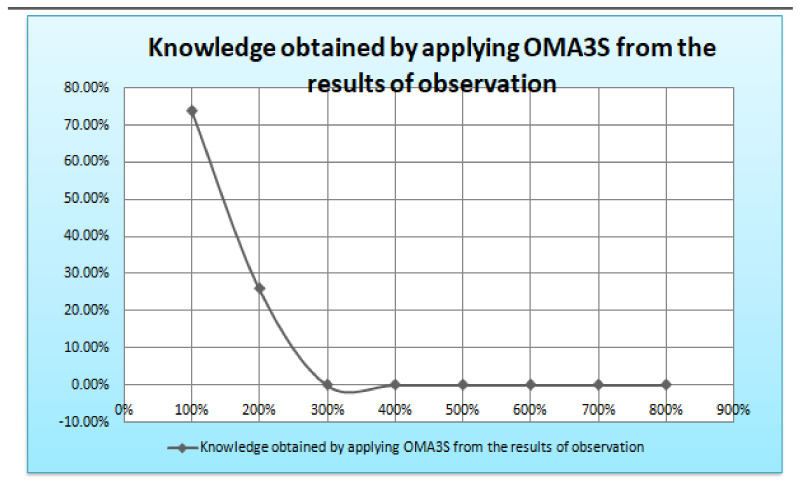
Atrial tachycardia tend to have 34.13% arrhythmia.

In Figure 9, the phrase “Atrial tachycardia, tend to have 34.13% arrhythmia” implies that those who have atrial tachycardia are more likely than not to also have arrhythmia in a 34.13% likelihood ratio. An aberrant heart rhythm known as atrial tachycardia is one particular form and is characterized by a fast pulse coming from the atria. The percentage might be an indicator of the observed prevalence or co-occurrence of arrhythmia in people with atrial tachycardia. Important to note is that this data may be from patient data, medical research, or statistical analysis of pertinent medical records. An interpretation can be given that is more accurate if more context or information is given.

In Figure 9, When a person is diagnosed with a left bundle branch block (LBBB), the phrase “Left bundle branch block having 18.76% LAD” implies that 18.76% of such people also have left anterior descending artery (LAD) involvement. A delay or obstruction in the electrical impulses travelling via the left bundle branch of the heart’s conduction system characterizes the cardiac conduction condition known as left bundle branch block [45]. The percentage may reflect the observed prevalence or incidence of LAD involvement in LBBB patients. The term “LAD” refers to a specific coronary artery. This knowledge may come from clinical observations, medical research, or statistical analysis of pertinent patient data [46].

**Figure 10:**
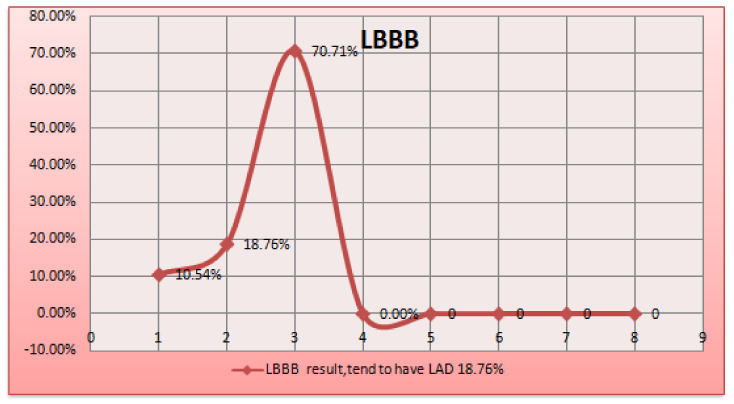
Left bundle branch block having 18.76% LAD.

**Figure 11:**
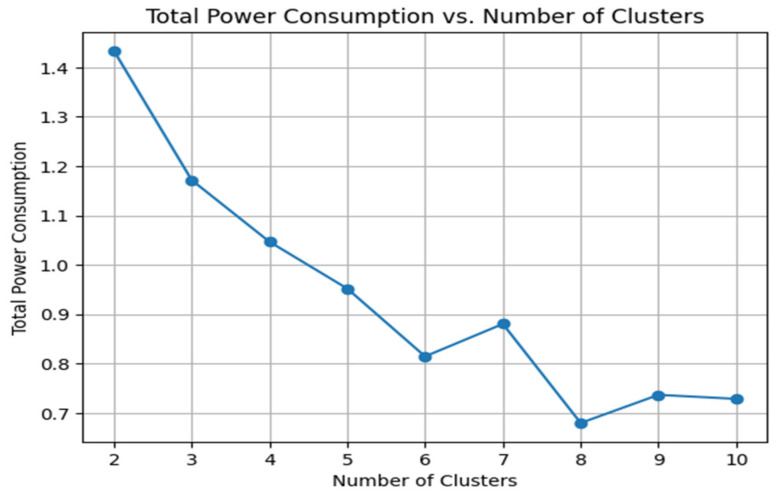

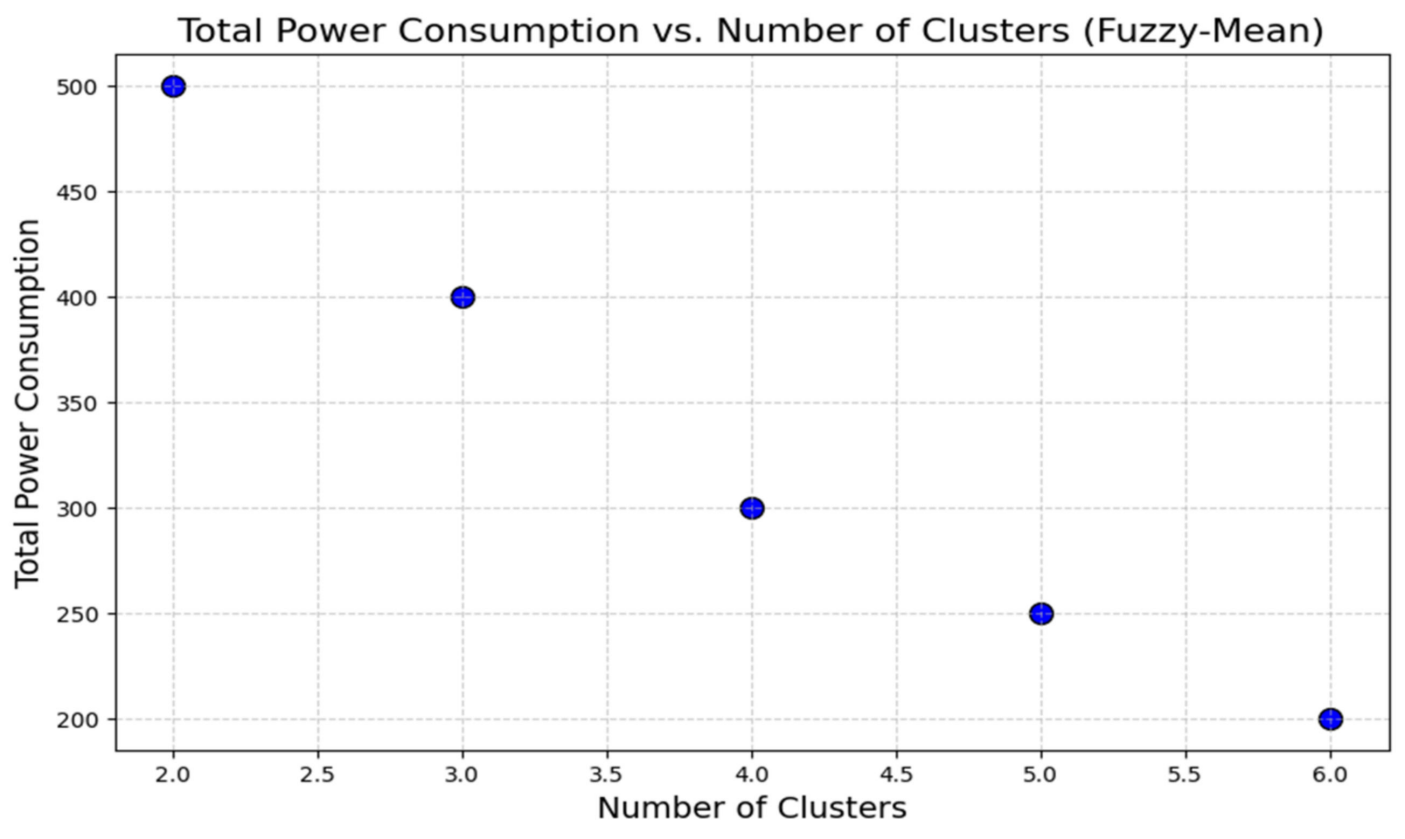
Data stream and sensing simulation results between total power consumption vs. number of cluster in fuzzy-mean.

We found a significant correlation between overall power usage and the number of clusters in the simulation results comparing data stream and sensing with fuzzy-mean clustering in Figure 10 [47]. The overall power consumption fluctuated as the number of clusters grew, demonstrating a dynamic interaction between clustering effectiveness and energy use. These results highlight the importance of cluster optimization in regulating power resources during data stream processing in a fuzzy-mean clustering setting. Further research is required to optimize cluster topologies for power efficiency in data sensing applications in Figure 11 [48].

### 4.1. DANA Algorithm

In accordance with their residual energy levels, nodes regularly activate. Nodes that are activated simultaneously create clusters. Let us say for the sake of simplicity that at time T0, nodes S1, S6, S11, S16, S21, S26,…, S46 are activated and form a cluster, and at time T1, nodes S2, S7, S12, S17, S22, S27,…, S47 are active and create another cluster. At the cluster heads (S2, S12, S22, S32, and S42), data would be pooled for the LEACH algorithm, and they would broadcast this aggregated data to the base station. According to the DANA algorithm, there would be two clusters of active nodes (at T0 and T1), and each cluster would send data to the base station.

**Figure 12:**
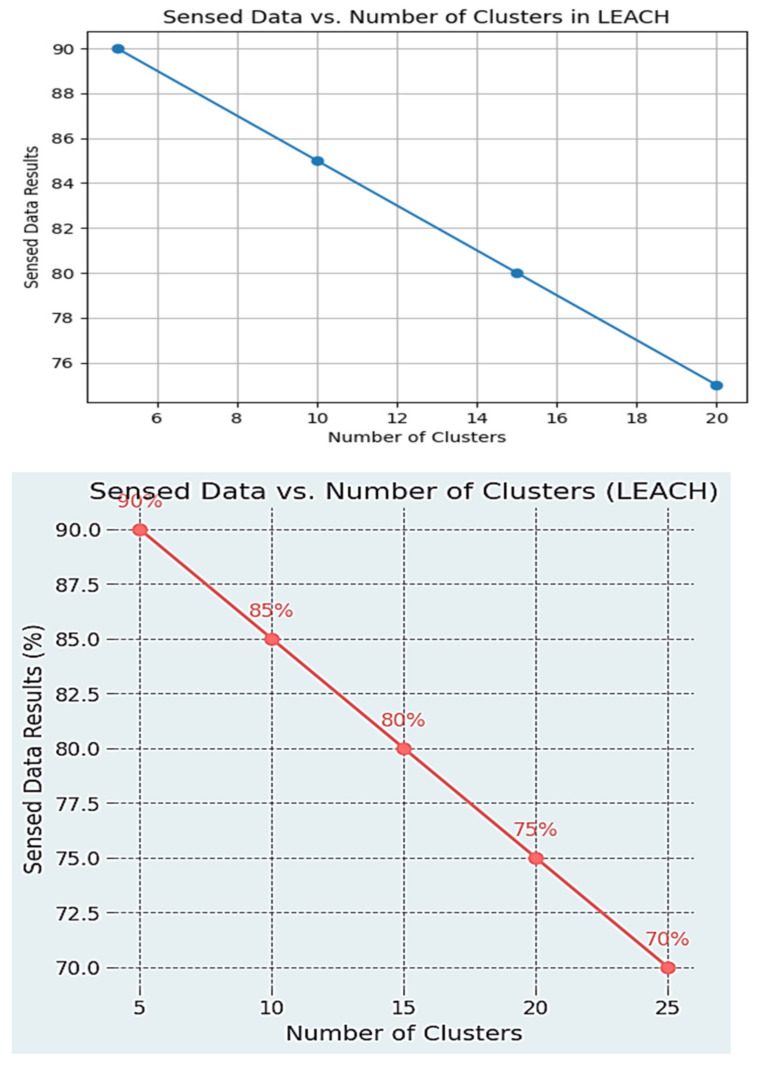
Data stream and sensing simulation results between sensed data vs. number of cluster in leach method.

The sensor network is in Figure 10, sensor network area consists of five locales with numbers 1 through 5. Each district has its distinct signal characteristics and is camouflaged differently. As a result, sensors nearby are determining results that are frequently identical to sign data. Let us assume there is a fundamental principle that governs the data connections between the sensor center points: at any given position, any two sensor data values are sufficient to aggregate the data potential advantages of any additional sensors in the vicinity. Each sensor center has hyperedge ((si, sj), s) events for all arrays of sensor centers si and sj with region locations comparable to s. m. Figure drill center method. By applying the above data-related rules, it is easy to see that the M sensor consists of about two centers from each location. This is sufficient to understand the code data for the various sensors in the sensor map. N (s, d) should be understood to refer to the sensor association’s correspondence outline for the s’s d-bob neighbors. Every sensor association center point should put together sufficient (let us say, K) models from every center in N (s, d), where d is big enough to gather all data relationships. This will enable every center point to lock in with every connecting edge. By periodically delivering data to the data collecting center point via a correspondence tree, starting at the beginning, when the association structure is dark, all of the association centers are progressively brought in. As a result, we can compile tests (K from each center) using data collecting messages for d reviews. Let us acknowledge that every center point has collected models from every I-hop neighbor. The inductive phase is, as was said earlier, during the (I + 1)st see, a center point v transmits the data collection message to its parent by (a) piggybacking the models of the I-hop neighbors it has proactively generated and (b) utilizing a transmission rather than a unicast transmission. Also recognized here are the models of v’s 1-bob and I-bounce neighbors. Since all of v’s 1-skip neighbors likewise wrap up the aforementioned piggybacking during its portrayal, which is comparable to testing from all of v’s (I) neighbors, the center point v would have acquired instances of all of its 1-bob neighbors’ 1-hop neighbors. In the previous cycle, we agreed that the I-bounce neighbors’ full model sizes are confined (because d should be as little as possible as a result of the local spatial data linkages), and may thus be packed or piggybacked in a single transmission. If the total models (K from each center) from the (d 1) bob region require x group messages, use the aforementioned piggybacking structure for xd reviews or add extra (at most ndx) messages. The method may be used to decrease the number of piggybacked portrayals required, as shown in Figure 12 [49] while the principal K models are being obtained. In Figure 11, it shows the use of cluster analysis by qualitative researchers can serve as a promising technique for theory- and context-informed interpretation. The methodology applied to the data collection platform (DCP) interviews produced fresh data on the qualitative coding and its interpretation. Its application in this case exemplifies a real mixed technique, in which a quantitative approach may support an analysis in which the qualitative data is crucial for the analytic outcome. The method offers researchers a systematic way to direct subgroup analysis within a qualitative data set, which opens up new avenues for qualitative study. In order to verify the correctness of suggested cluster solutions, we underline that the clustering approach we have discussed necessitates integrating various methodologies. Cluster analysis does not offer definitive guidelines for decision-making; it merely offers advice in the form of suggested configurations [50].

In Figure 12, clustering and data transfer phases are included in each cycle. The top 10 CHs are chosen for the clustering phase and group together according to Euclidean distance. The CHs are chosen for every cycle until all of the nodes have used up all of their energy. The base station is situated distant from the field. The number of cycles it takes for the first node in the network to exhaust all of its energy is used to calculate the network’s lifespan.

**Figure 13:**
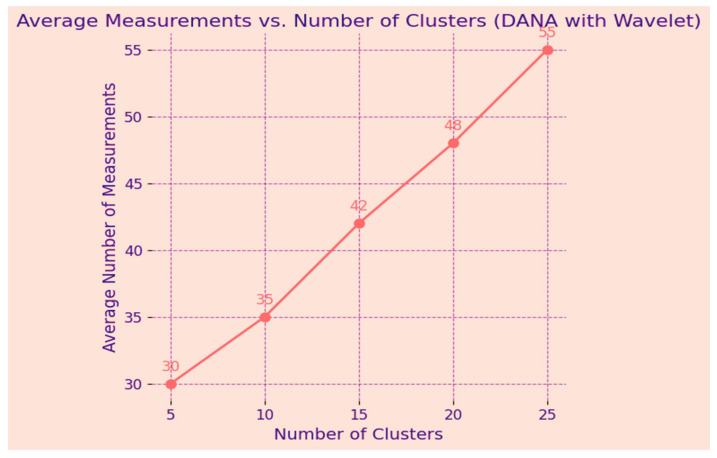
Data stream and sensing simulation results between average number of measurement vs. number of cluster using wavelet in DANA method.

In Figure 13, the number of clutters in *y*-axis, and *x*-axis illustrates the outcomes of the experiment, in which sensor nodes are distributed at random over a square area of m2, and network lifespan is displayed, which displays the number of living nodes with time in cycles. Finally, the outcomes are contrasted with a well-known DHAC technique. According to reports, DHAC is more energy-efficient than other processes, such as LEACH [51].

**Figure 14:**
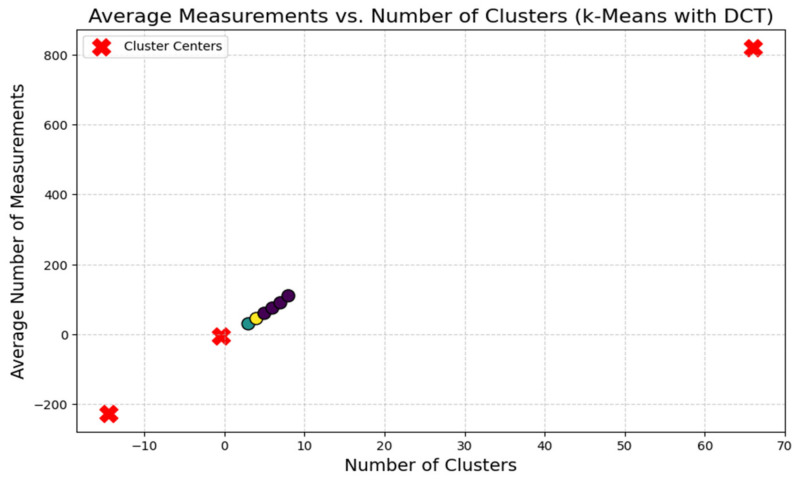
Data stream and sensing simulation results between average number of measurement vs. number of cluster using DCT in k-MEAN method.

In Figure 14, Networks with hierarchical topologies can provide QoS using k-means. K-means demonstrated a delay reduction of 91.72% in the random situation and 95.39% in the deterministic scenario when compared to LEACH. K-means showed an 81% decrease for the random situation and a 97% reduction for the deterministic scenario in terms of fuzzy [52].

**Figure 15:**
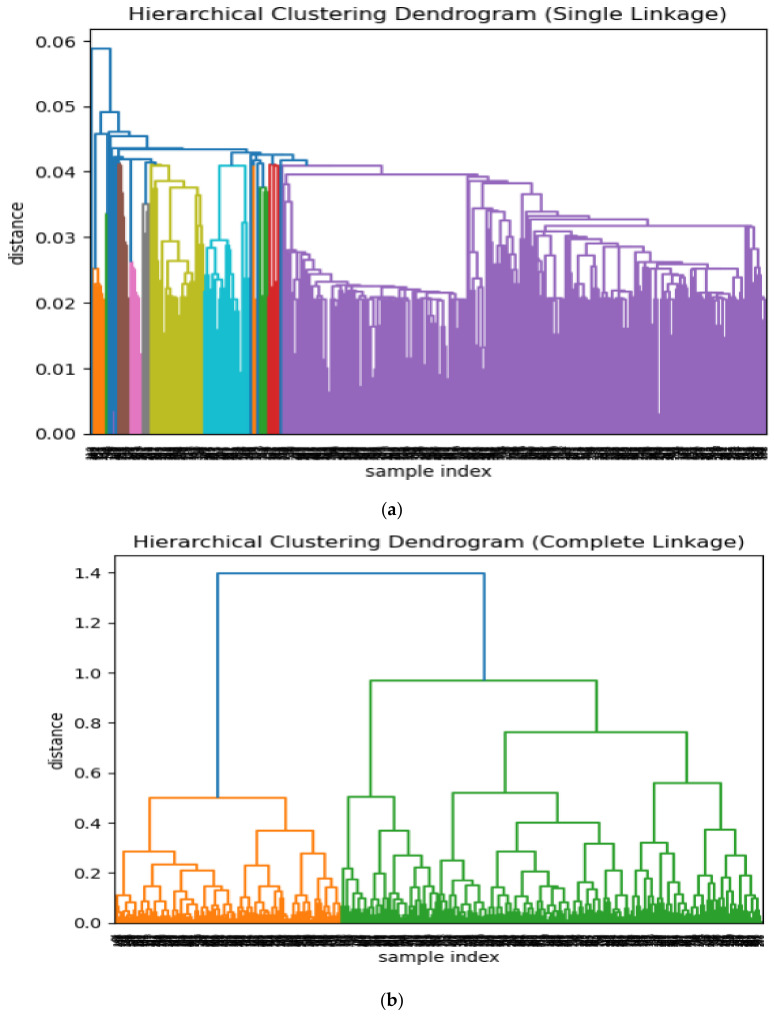

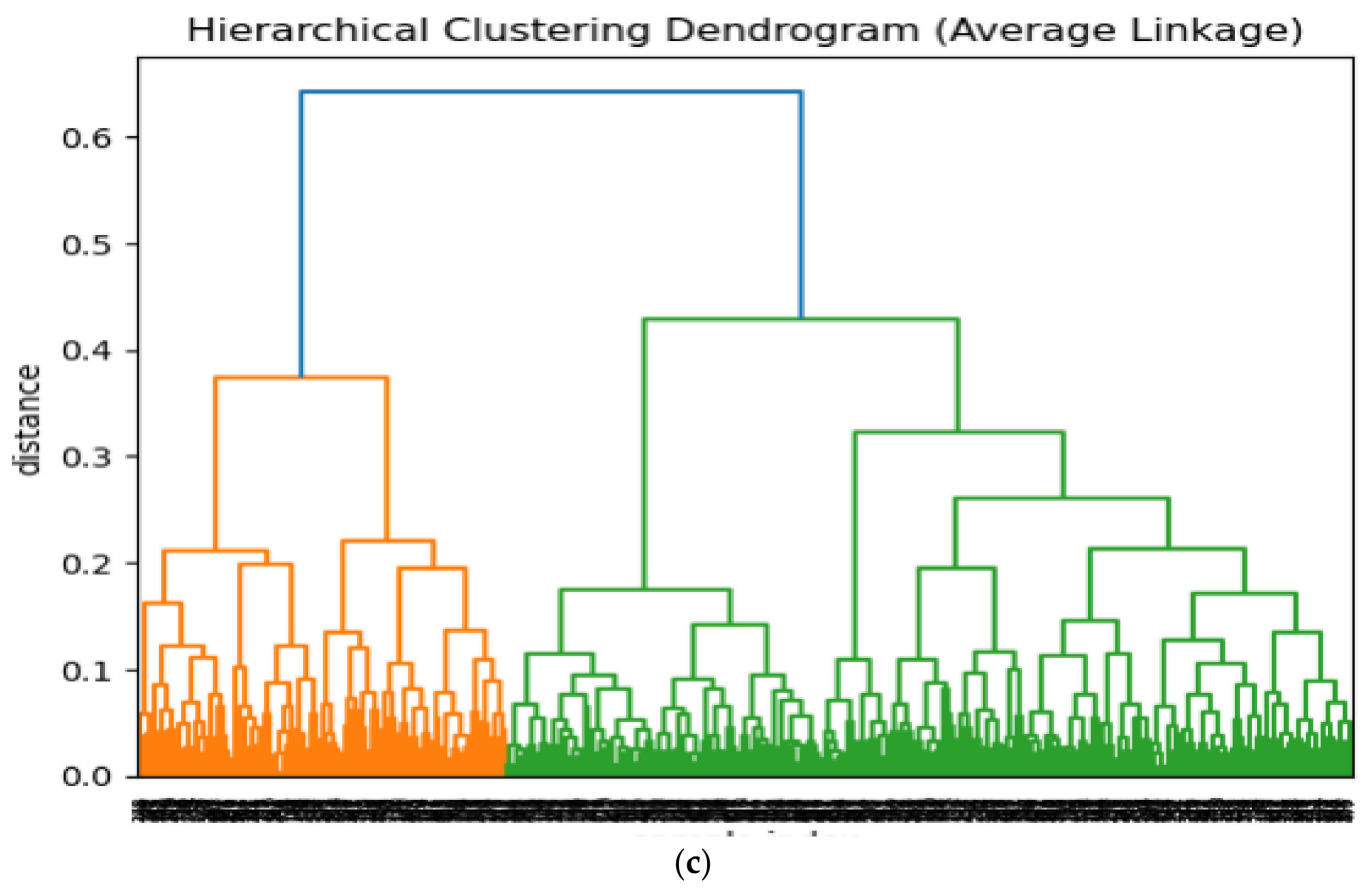
Correlation between data in efficient optimization of condition for marking deleted of a node s. (**a**) The condition C2 involves communication edges using dendrogram, (**b**) while C3 and C4 involve correlation edges complete linkage. (**c**) Correlation graph and its intersection graph of source sets average linkage.

The simulation results displayed in this section demonstrate the applicability of our suggested methodology and computations. Our suggested diffused and concentrated computations, including the 2-Rounds, Handshake, 0-bounce, 1-jump, and 2-jump concentrated algorithms, are compared for the size of the related relationship overwhelming set they each produce. Recognize that 0-bounce is primarily a credulous, avaricious, and targeted strategy. The communication expenses incurred by the two scattered estimates are also taken into consideration. We fail to account for the communication costs associated with acquiring neighborhood data and doing data testing (for calculation of connection edges), since they can be assembled utilizing the piggybacking system or will cause practically the same expense for every single appropriated approach. Note that because of the little upsides of d, k, and K (2, 2, and 3 separately) utilized in our recreations, the piggybacking methodology is required over just a few previews, as shown in Figure 15a [53].

The hard and quick amount of messages caused and the save reserves gained (b) in Figure 15b leads us to forecast that the approach of nurturing an association overpowering set utilizing the 2-Rounds computation will generate massive energy hold reserves as indicated in Figure 11. In Figure 15a, A preliminary definition of cluster centers is necessary for K-means. The approach picks a variable whose mean is utilized as a threshold for separating the data into two clusters, starting with one cluster. The initialization of k-means is then done using the cancroids of these two portions in order to maximize the membership of the two clusters. Next, one of the two clusters is chosen for splitting, and a variable inside that cluster is picked whose mean is utilized as a threshold for dividing that cluster in two. The data are then divided into three clusters using K-means, which are started using the centric of the surviving cluster and the cancroids of the two split cluster sections. Up until a predetermined number of clusters are found, this process is repeated [54]. In Figure 15b, By taking original data reconstruction quality into consideration, the right sample rate should be chosen. Here, DANA was used to assess reconstruction quality as it is one of the crucial variables for selecting the right sample rates in real-world applications. The gap between anticipated and actual switching times for all mode alternations was averaged in order to measure the switching perception delay with the goal of evaluating switching performance. When actual switching behavior happened later than anticipated, the time difference for each mode change from prediction-based to control-state modes was recorded as positive. In Figure 15c, the strength of the association between two variables is gauged by correlation coefficients. When two variables are correlated, it may be seen in a model for WSN that when one variable’s value changes, the other one tends to change in a particular way. For instance, weight and height are connected; as height rises, so does weight inclination. Therefore, if someone is noticeably taller than average, we can assume that he is likewise heavier than typical [55].

**Figure 16:**
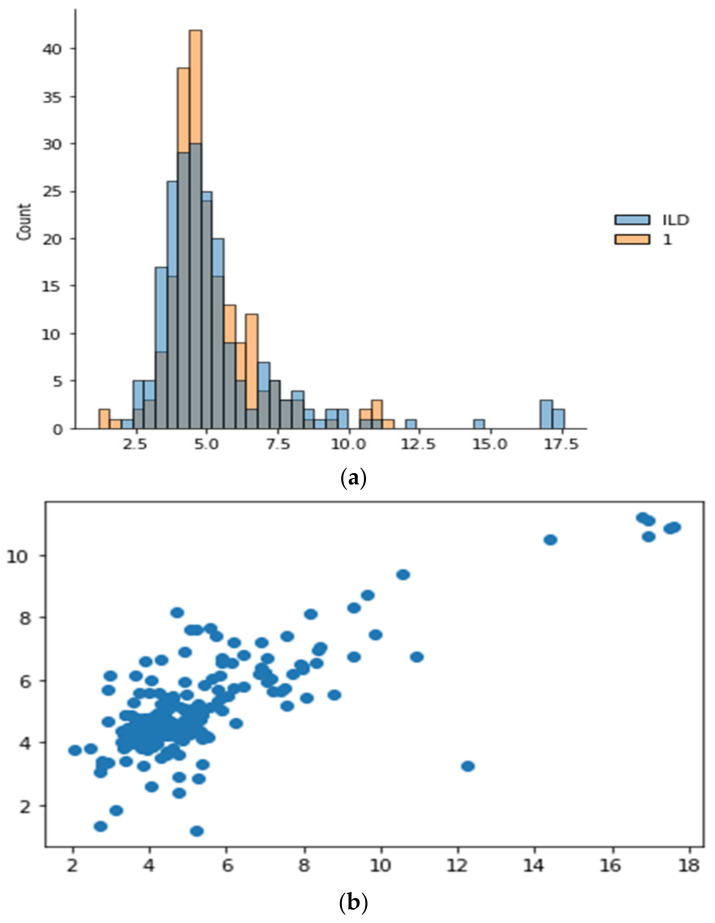

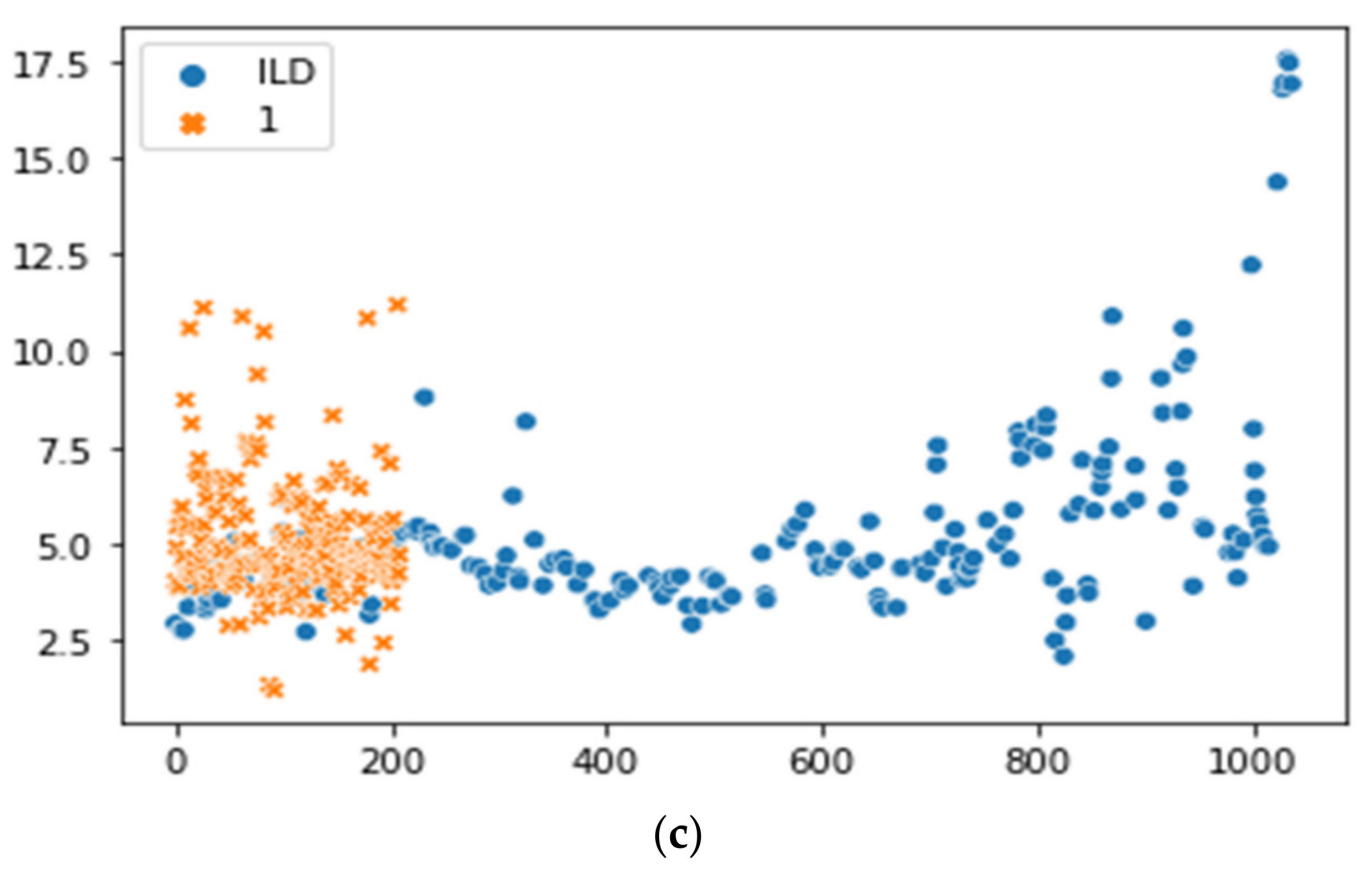
Data allocation techniques showing in Figure (**a**–**c**).

In Figure 16, the *y*-axis displays the time it took to cluster the datasets in seconds, while the *x*-axis displays the number of virtual machines used. The outcomes show how the suggested method outperforms the conventional approach when used with various settings. Another metric used to compare the two methods is the execution time. With regard to Hadoop’s features, we have sped up the clustering reaction time. For the same collection of data items, the impact of the number of nodes employed on the clustering techniques was also investigated. Scalability levels were evaluated by increasing the number of execution nodes while keeping the same datasets and the same number of nodes while changing the dataset sizes. In Figure 16a, we visited the manually measured ground truth points in 7.13 (a). Despite the way the doors and edges are made, there is laser scan ambiguity in the test setting. These components cannot be assessed due to the narrow sensor location range (in our studies, roughly 4 m), therefore the location instead gathers uniform data from the wall of targets and locations. In Figure 16b, the overall location probabilities for all the faults, each of which represents the likelihood that no sensors would sound the alarm for the associated fault, should be minimized if we take into account all the locations The location probability of the system is equal to the sum of all faults, under the assumption that there is only one problem in the system. This assumption is reasonable given the extremely low likelihood of many faults occurring at the same time. Consequently, we have the following optimization challenge.

Let us imagine that we have 50 sensors (S1 to S50) set up in a 100 m × 100 m field, and they continuously produce temperature data. I will give a random sample of data points for this example as follows:

Let us now organize the sensors into groups and combine their data using the LEACH and DANA clustering methods.

### 4.2. LEACH Algorithm

1. The initial probability P that each node will become the cluster head should be determined during setup.
2. Cluster Formation: Choose the cluster leaders for the current round according to probability P.
3. Data collection and aggregation: The cluster leaders gather data from their cluster members.
4. Changes to the Round: To allocate the energy usage equally for the new round, recalculate the probability.
5. S2, S12, S22, S32, and S42 will be used as the cluster heads in the first round for the sake of simplicity.

**Figure 17:**
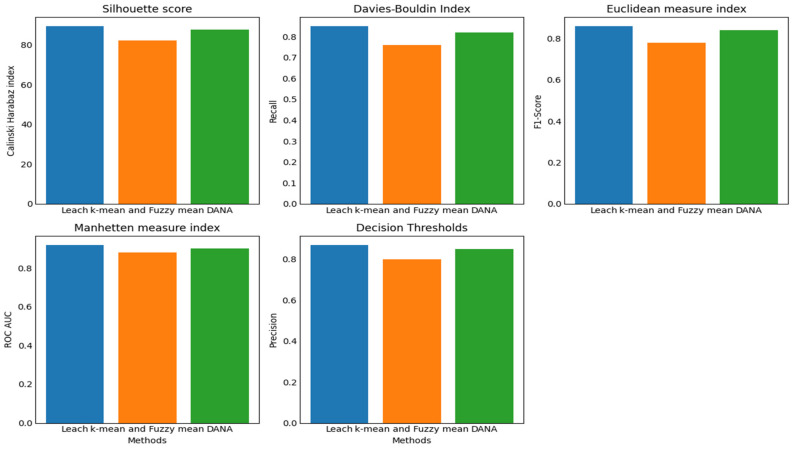
Comparison of all proposed method in terms of Silhouette score, Calinski–Harabaz index, Davies-Bouldin Index, Decision Thresholds, Manhattan distance, and Euclidean distance performance on different indexes.

We may construct a hypothetical dataset with six parameters for every sensor node to provide a table for the LEACH and DANA clustering methods. For each node, let us posit the following details: Every sensor node’s Node_ID serves as a distinctive identification. X-coordinate: The sensor nodes exact position in the field as indicated by its X coordinate. Y-coordinate: Y-coordinate of the sensor node’s location in the field. Temperature: The sensor node’s output of the temperature measurement. If a node is a cluster head or not, Cluster Head returns 1 (if it is, else it returns 0). A node’s activation time for data transmission (just for DANA) is known as activation time. Each algorithm (LEACH and DANA) is represented by two rows in this table, and each algorithm’s corresponding percentage result is displayed in a separate column. Please be aware that the percentages displayed here are only estimates and that the final results should be determined by your unique examination and analysis of the data for each method. The percentages are some type of assessment meter or performance measure for the individual algorithms based on the supplied data, and they may be used to evaluate how well the algorithms perform in terms of fulfilling their intended goals in the scenario. Figure 17 shows the compassion of all proposed methods in terms of Silhouette score, Calinski–Harabaz index, Davies–Bouldin Index, Decision Thresholds, Manhattan distance, and Euclidean distance.

**Figure 18:**
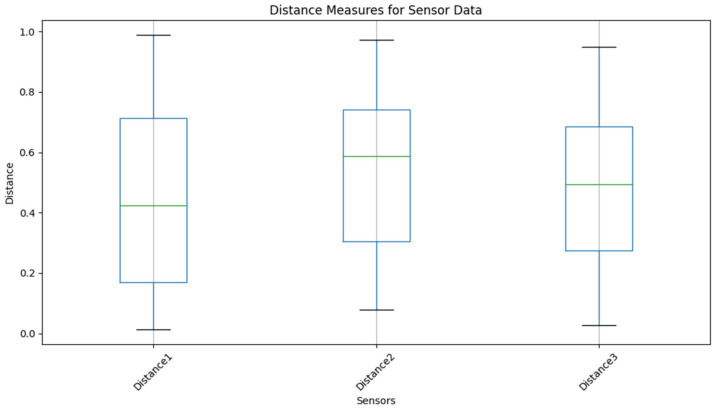
Sample sensor data and corresponding distance measures.

In Figure 18, we first build a sample dataset using sensor data and related distance measurements. Replace the sample data with your own sensor data and estimated distances. The distribution of distance measurements for each sensor is then depicted using a box plot created using matplotlib and pandas from a data frame created from the data.

**Figure 19:**
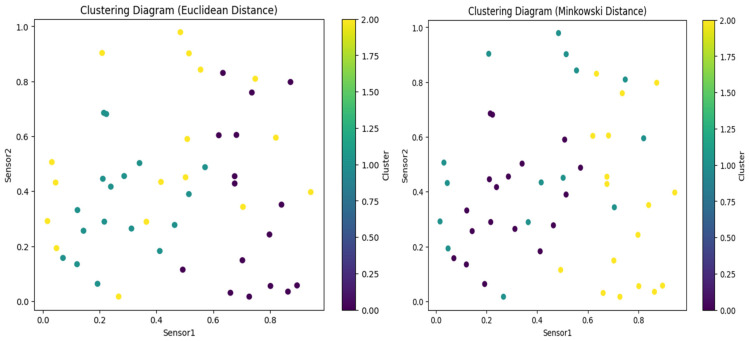

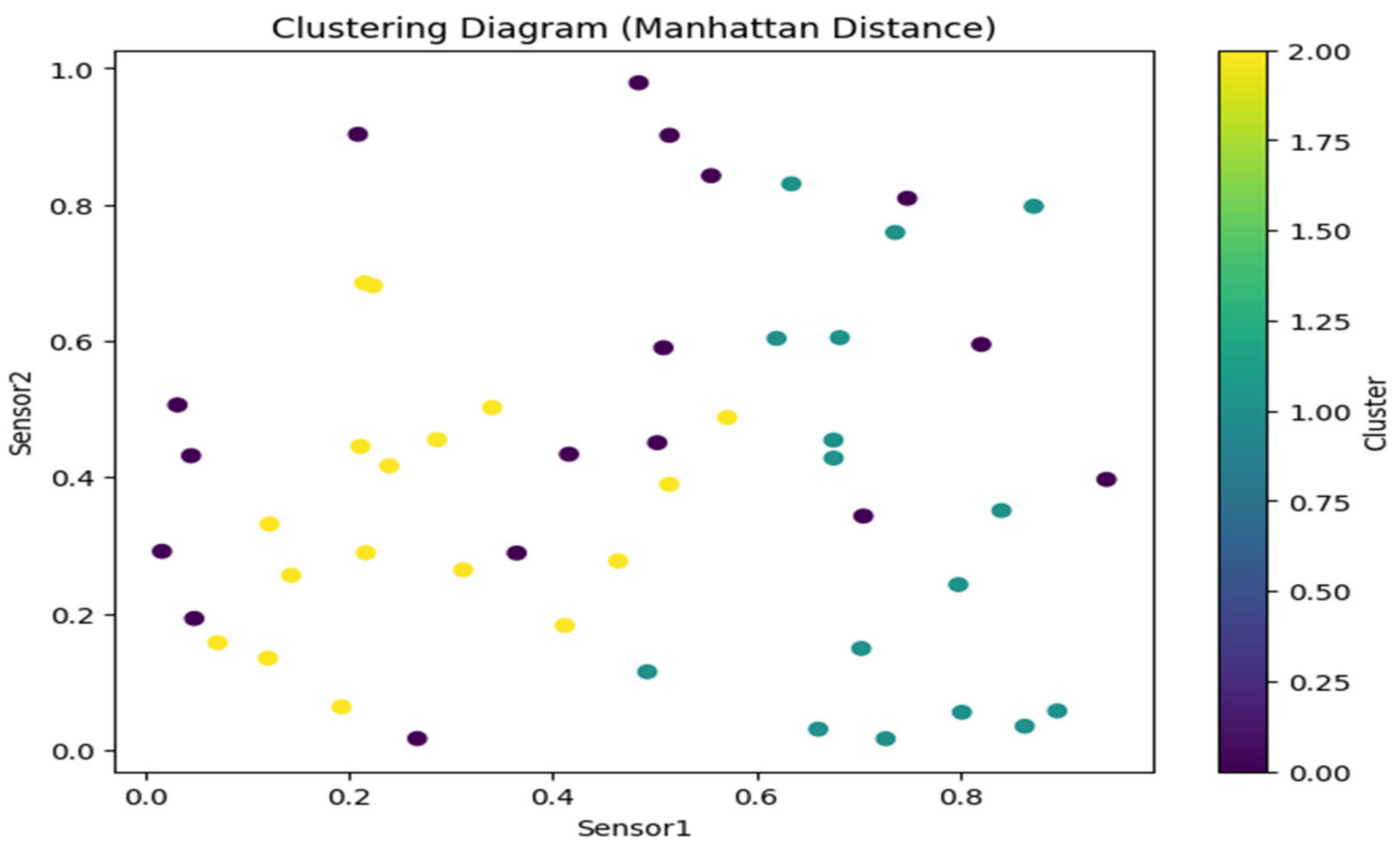
Comparison plot of all three distance measures for grouping sensor data.

**Figure 20:**
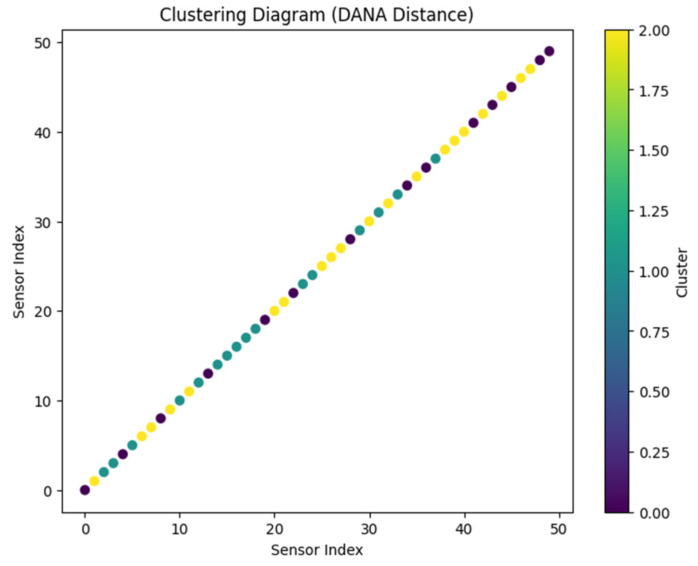
Plot of DANA distance measure clustering algorithm on input data.

**Figure 21:**
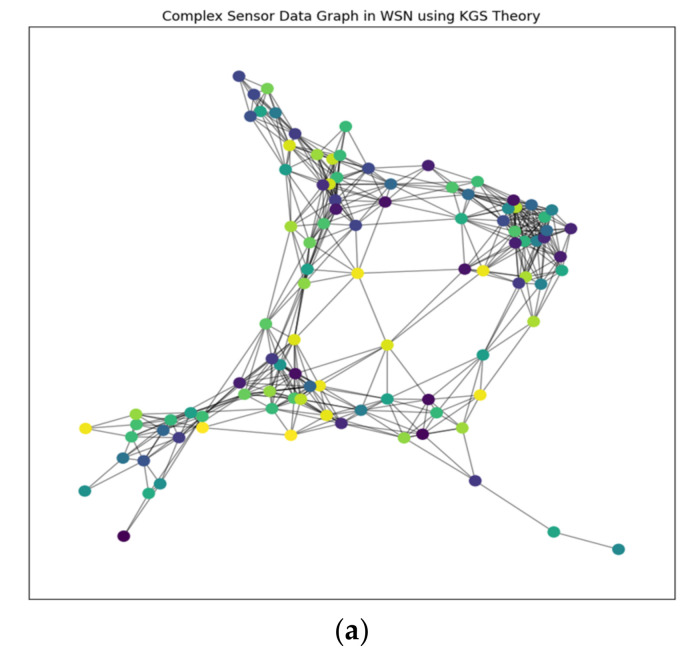

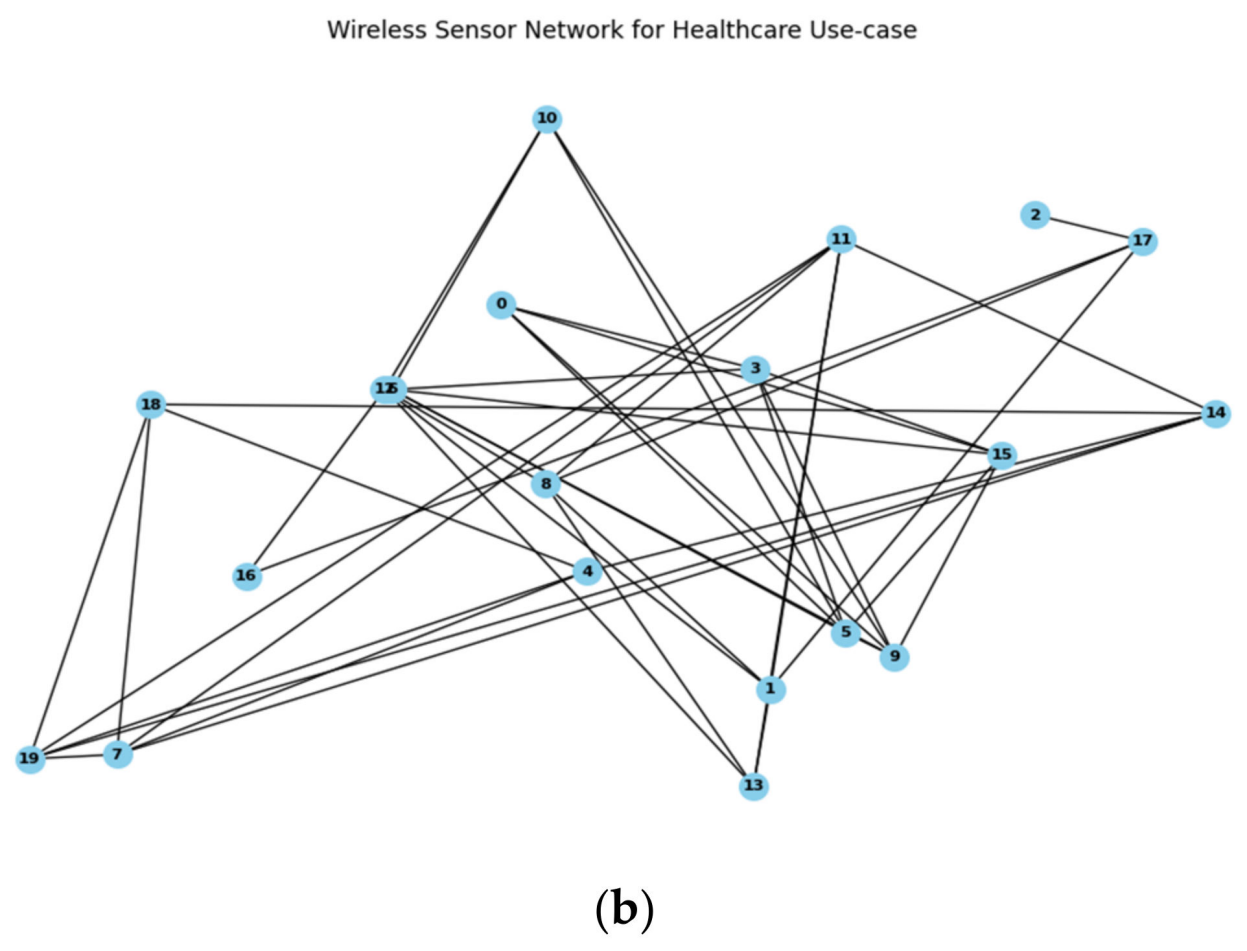
(**a**) Representation of complex sensor data node in KGS THEORY. (**b**): complete wireless sensor network (WSN) for signal processing-based disease detection in a healthcare use-case.

In Figure 19, This code starts by creating an example dataset with sensor data and a data frame to hold it. The Manhattan distance, Murkowski distance, and Euclidean distance metrics are then used to generate distance matrices. We run clustering using KMeans with a predetermined number of clusters for each distance metric, and then we make a clustering diagram to display the outcomes. In Figure 20, calculating the DANA distance matrix, executing clustering, and displaying the outcomes are all processes in the implementation of a clustering diagram for the DANA (dynamic adaptive network analysis) algorithm distance matrix. While DANA may be used to compute distance matrices, it is most frequently utilized in WSN for routing and network optimization. Your unique use case may need that you modify the method in order to build a clustering diagram. In this example, we assume you have a DANA distance matrix. We then use KMeans clustering to produce a clustering diagram based on that matrix. Put details real DANA distance information in lieu of the DANA_distance_matrix variable. In Figure 21a, to mimic the topology of the WSN, make a random geometric graph. The KGS model’s radius and number of nodes are determined, respectively, by the num_nodes and num_nodes parameters. For every node, model sensor data. Use genuine sensor data from your device instead, or a more accurate simulation, in its place. To see the data shown on the graph, create a color map for the nodes’ colors depending on the sensor data values. For a representation that looks good, arrange nodes using a spring arrangement. Using Matplotlib and Network, produce the complicated graph visualization. In Figure 21b, it takes more work than one code sample to build a comprehensive wireless sensor network (WSN) for signal processing-based disease detection in a healthcare use-case. I can provide you a streamlined illustration of a sensor network and a graph visualization to begin with, though. You would have to take into account a number of things in a real-world scenario, including data gathering, processing, disease detection methods, and more. Here is a condensed Python example that uses NetworkX to build a simple sensor network graph.

**Figure 22:**
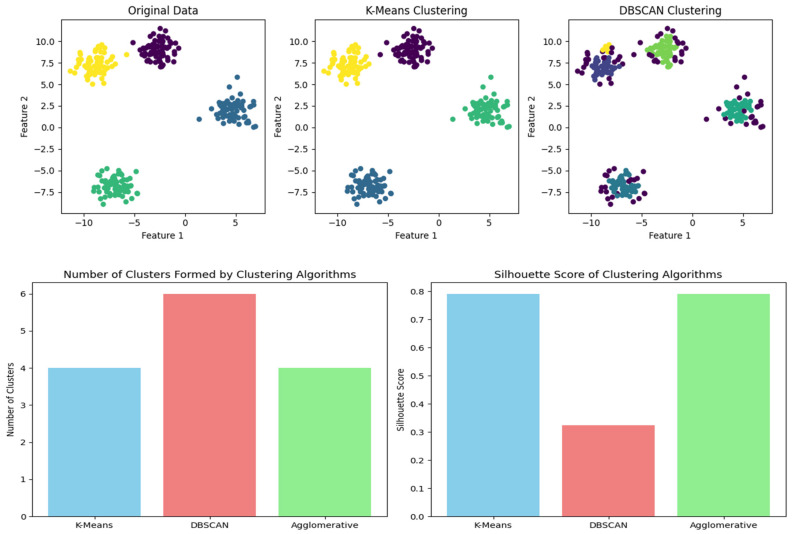
Different clustering methods for enhanced feature selection in data gathering.

In Figure 22, the process of putting various clustering methods for WSN in healthcare into practice is shown in the Python example I gave previously. The major steps in the example are broken down as follows: Utilizing the make_blobs function from the sklearn datasets package, we create fictitious sensor data in this example. For the actual sensor data that is often gathered from sensors in a WSN installed in a healthcare context, this synthetic data acts as a stand-in. Import Crucial Libraries: We import Crucial Libraries, such as Numpy for Numerical Operations, Matplotlib for Data Visualization, and Scikit-Learn’s KMeans, DBSCAN, Agglomerative Clustering, and make_blobs for Clustering and synthetic data generation. Synthetic Data Production: Synthetic data production is done using the make_blobs function. Realistically, you would swap out.

**Figure 23:**
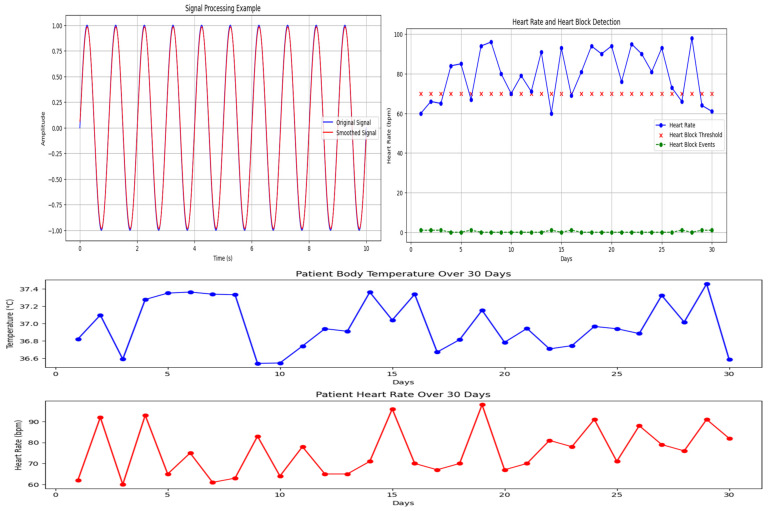
Heart blockage detection model enhancement result using enhanced data gathering technique using DANA method.

**Figure 24:**
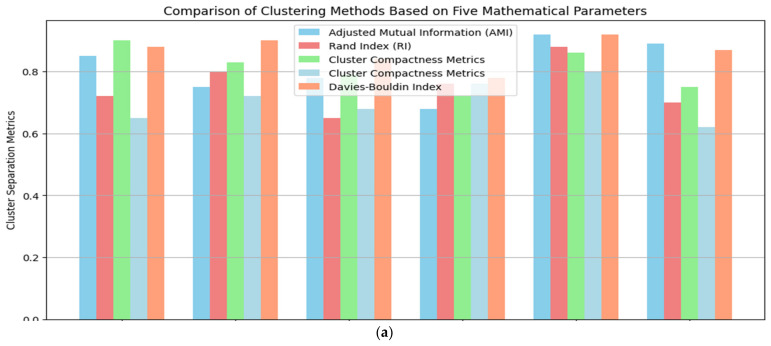

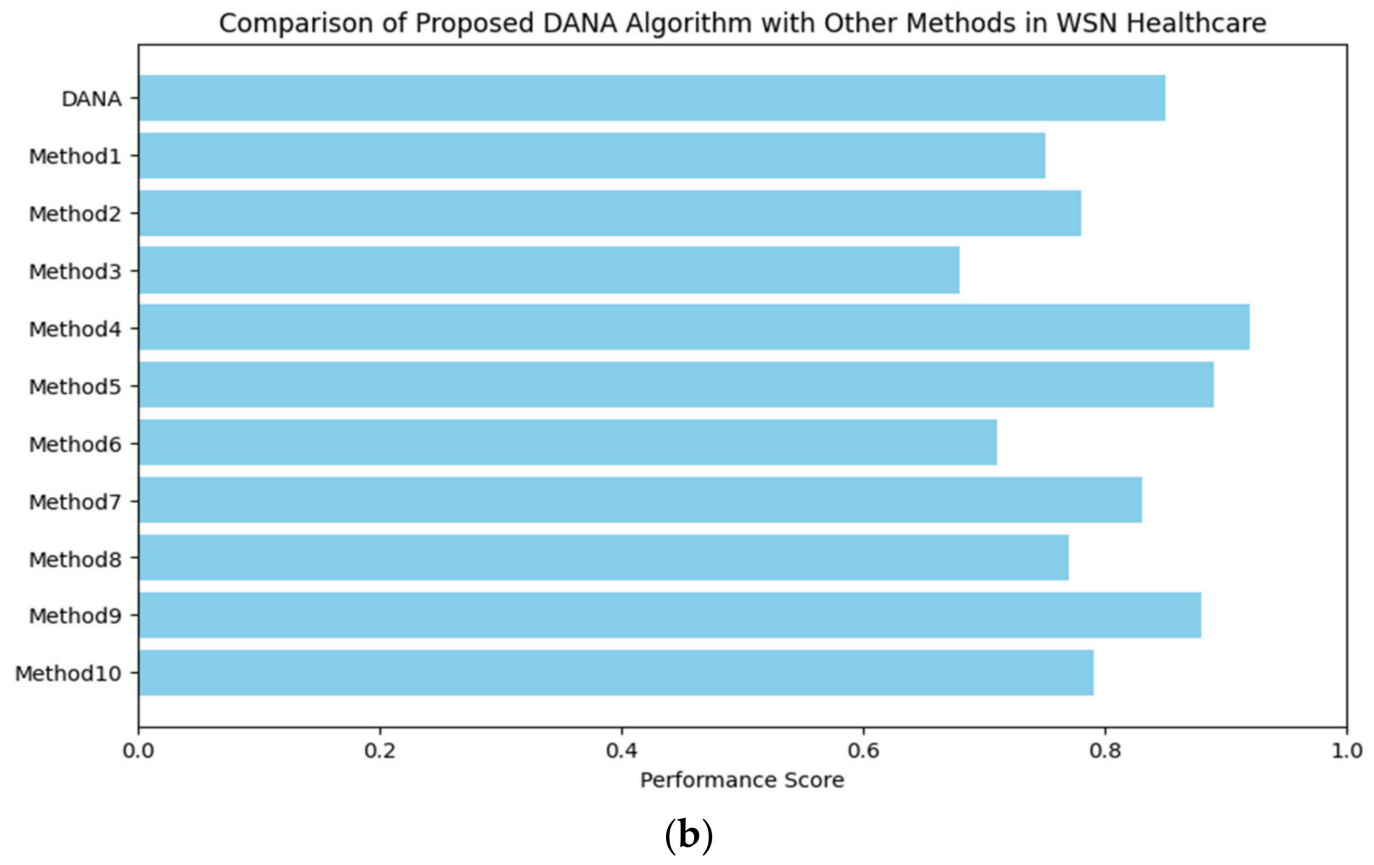
(**a**,**b**):Comparison graph of proposed model with other method. In (**a**) it shows references [8,11,14,22] compared with proposed one which shows in first bar.

In Figure 23, electrocardiogram (ECG or EKG) signal analysis is a crucial step in the diagnosis and treatment of cardiac problems and includes the identification of heart blocks. The appearance of heart blocks on an ECG signal implies interference with the heart’s normal electrical conduction, which is seen through the ECG as a window into the electrical activity of the organ. The gathering of ECG data is the first step in the heart block detection procedure. Typically, electrodes are applied to the patient’s skin at precise areas to record the electrical impulses produced by the heart. In order to interpret and analyze these signals, they are subsequently sent to an ECG equipment. First-degree heart block is typically characterized by a prolonged PR interval, which signifies a delay in the electrical impulse traveling from the atria to the ventricles. This delay is often asymptomatic and may not require immediate intervention. Second-degree heart block presents as intermittent failures in the conduction of electrical impulses from the atria to the ventricles. It can be further categorized into two types: Mobitz Type I (Wenckebach) and Mobitz Type II. Mobitz Type I is marked by a progressively lengthening PR interval until a beat is dropped, while Mobitz Type II involves random dropped beats without PR interval changes. Mobitz Type II is of particular concern as it can progress to a complete heart. Several preprocessing processes are frequently applied to the raw ECG data after it has been obtained to improve its quality. Filtering is employed here to eliminate noise that was a prolonged PR interval, which denotes a lag in the passage of the electrical impulse from the atria to the ventricles, is often the hallmark of first-degree heart block. Since this delay frequently has no symptoms, it might not need quick attention. Intermittent failures in electrical impulse conduction from the atria to the ventricles are the hallmarks of second-degree heart block. It can also be divided into Mobitz Type I (Wenckebach) and Mobitz Type II. In Mobitz Type I, the PR interval gradually gets longer until a beat is dropped, but in Mobitz Type II, beats are dropped at random without changing the PR interval. Mobitz Type II is very dangerous since it can develop into a total heart block. Third-degree heart block, also known as a complete heart block, is the most severe form. In this condition, there is a complete dissociation between the atria and ventricles, resulting in two separate pacemakers. The atria continue to beat at their own rate, while the ventricles beat at their own, often slower, rate. To detect these heart blocks, clinicians closely examine the ECG waveform for irregularities in the PR interval and the relationship between the P-waves and QRS complexes. It involves the acquisition, preprocessing, and visualization of ECG data, with a focus on identifying deviations in the PR interval and waveform patterns associated with heart block. Early detection and accurate diagnosis are essential for appropriate medical intervention and the effective management of cardiac conditions. In Figure 24, the comparison of proposed clustering method is compared with the other work as given in references serially. Scores for metrics such as the Davies–Bouldin Index, the Rand Index, the Cluster Compactness Metrics, and the Adjusted Mutual Information (AMI) are visualized for each approach, allowing for a quick and insightful comparison of the clustering results obtained using each method [55].

Wireless sensor networks (WSNs) are increasingly becoming vital components in the healthcare domain, offering unparalleled opportunities for monitoring, data collection, and patient care. The integration of WSNs in healthcare not only ensures real-time data acquisition but also enhances the precision and efficiency of medical procedures and patient monitoring [54]. The manuscript in question delves into this subject, addressing key aspects and potential applications of WSNs within the healthcare sector. Given the critical nature of healthcare applications, the relevance of this work cannot be overstated. It has the potential to significantly contribute to the ongoing discourse and development in the field, fostering a better understanding and adoption of WSNs in medical practices. The manuscript highlights the transformative impact that WSNs can have on healthcare, especially when combined with intelligent algorithmic decision-making. However, to unlock its full potential and ensure that it effectively reaches and resonates with its intended audience, the manuscript necessitates careful and thorough editing [55]. This will not only enhance its clarity and coherence but also ensure that the technical nuances and innovations are accurately and compellingly presented. With these refinements, the manuscript is poised to become a valuable resource, aiding in the seamless integration of WSNs in healthcare and ultimately contributing to the betterment of patient care and medical practices [56].

In summary, the semi-supervised clustering-based DANA algorithm for data gathering and disease detection in healthcare WSNs shows promise in terms of its methodological approach; however, in order to make it practical to use, it will need to address issues related to scalability, resource consumption, adaptability to changing environments, strengthening security measures, accommodating heterogeneity in sensor nodes, and giving priority to user-friendly implementations. In addition, the algorithm’s efficacy, repeatability, and ability to support continuous improvement depend heavily on thorough validation, benchmarking, and the promotion of transparent cooperation [57]. By taking these factors into account when developing the algorithm, the algorithm’s applicability and effect in real-world healthcare settings will be increased, bringing methodological developments into line with complete and long-term solutions for illness detection via wireless sensor networks [58,59].

## 5. Conclusions and Future Scope

The semi-supervised clustering-based model, coupled with the DANA algorithm, has demonstrated significant potential in disease diagnosis within wireless sensor networks (WSNs) for healthcare applications. The DANA algorithm efficiently minimizes energy consumption and communication overhead by enabling dynamic, adaptive routing. This model’s integration of both labeled and unlabeled data enhances disease identification accuracy, even with limited labeled samples. Proposed work specifically explores various data collection methods and sensing techniques in extreme wireless sensor networks. We have shown how different network types can be adapted for diverse data collection strategies using signal processing. Additionally, we haveinvestigated various clustering-based methods, highlighting the high accuracy of K-mean clustering and fuzzy set interference, aligned with big data parameters like volume, diversity, and velocity. Advanced strategies have been proposed for event out-routing and big data algorithms. Proposed approach significantly reduces cluster distance calculations, offering an evidence-based data collection method. We have demonstrated multiple use cases, particularly in measuring ECG and EEG characteristics with various sensors. Our research has also introduced new software techniques, including the use of a sliding window method for data stream management optimization and a novel treatment algorithm for clustering processing streams. Furthermore, we propose a real-time sensing model, the DANA model, suitable for big data and string-based data collection methods.

Although the DANA algorithm for semi-supervised clustering-based data collection and disease detection in healthcare WSNs is a promising method, there are a few things to keep in mind. As networks grow in size, scalability problems might appear and optimization techniques would need to be looked at. For sensor nodes to have longer lifespans, any resource consumption issues must be resolved. Practical deployment requires a number of factors, including user-friendliness, accepting heterogeneity in sensor node configurations, improving security measures for patient data, and guaranteeing strength in dynamic contexts. Establishing the algorithm’s effectiveness, promoting repeatability, and enabling ongoing progress need extensive validation, benchmarking, and transparent cooperation. Enhancing the algorithm’s relevance and influence in real-world healthcare settings should be the top priority for researchers.

## Figures and Tables

**Table 1:** Comparative study of several state of the art methods using different methods.

Approaches	Attributes of Energy Efficient Gathering						
	Hetrogeniousity	Mobility	Space Complexity	Memory Usage	Length of Input Data	Clustering Ojective	Big Data Parameters Like Volume, Veracty Effiency…
[2]	🗸	no	🗸	Avg.	🗸	Energy consumption	no
[12]	🗸	no		High	🗸	Scheduling	no
[21]	🗸	🗸	🗸	High	🗸	Load balancing	no
[11]	🗸	🗸		High	🗸	Throughput	no
[17]	🗸	🗸	🗸	High		Packet delivery	🗸
[24]	🗸	🗸			🗸	Data storage	🗸
[13]	no	no	🗸	Efficient	🗸	Processing	🗸
[5]	no	no		🗸		Warehousing	no
[29]	no	🗸	🗸	Avg.	🗸	Shrinkage	🗸
[30]	🗸	no		🗸	🗸	Analytics	🗸
Proposed	🗸	🗸	🗸	Efficient	🗸	Overhead reduction, reduction of low latancy interference, storage, energy efficient data gathering, analytics	🗸

**Table 2:** Correlation hypothesis and ECG observation.

Hypothesis	ECG Observation
Baseline Norm	Lead V6 shows an R wave in a positive direction.
Standard Norm	Lead V1 shows an R wave in a positive direction.
Left Heart Orientation Shift	Lead I show an R wave in a positive direction, and avF shows an R wave in a negative direction.
Regular Cardiac Rhythm	ECG displays a normal heartbeat.
Right Heart Orientation Shift	Lead I show an R wave in a negative direction, and avF shows an R wave in a negative direction.
Normal Cardiac Conduction	R wave is present in lead I and avF.
Right Bundle Block (RBBB)	Lead V6 shows S amplitude less than 0.1 mV and S interval greater than 80 ms.
Bundle Branch Block (BBB)	Lead VI displays a QRS interval greater than 20 ms.
Right Bundle Block (RBBB)	V3 and T waves appear to merge together.
Left Bundle Block (LBBB)	ST elevation observed in lead V4.
Left Bundle Block (LBBB)	Lead V5 displays Q amplitude less than 0.1 mV.
Cardiac Arrhythmia	ECG indicates a slow heartbeat.
Cardiac Arrhythmia	In one lead, there are changes in the break of RR intervals.
Atrial Tachycardia	Is electric baseline observed.
Atrial Tachycardia	Abnormal P wave pattern.
Ventricular Tachycardia	ECG shows RSR’ complexes with taller R waves.
Ventricular Tachycardia	P and QRS complexes appear at different rates.

**Table 3:** List of Hypotheses for CAI Software (version 1), according to Table 1.

NO	Observations
1	R wave in lead I+ and R wave in lead avF+
2	V1
3	V2
4	V3
5	V4
6	V5
7	V6
8	Wide S wave in lead V1
9	Merging of T and-in lead V4
10	Merging of T and 9 in lead V3
11	V5, Q amplitude < 0.1 mV

**Table 4:** Correlation hypothesis observation.

Time	Cluster	Distance Measures					Fuzzy	Leach	K-Mean	DANA
		1	2	3	4	5				
T1	K1	1	1	1	1	0	0.8			
	K2	0	0	0	0	1	0.2			
	K3	0	0	0	0	0	0			
	K4	0	0	0	0	0	0			
	K5	0	0	0	0	0	0			
	K6	0	0	0	0	0	0			
	K7	0	0	0	0	0	0			
	K8	0	0	0	0	0	0			
T2	K1	1	1	1	0	0		0.6		
	K2	0	0	0	1	1		0.4		
	K3	0	0	0	0	0		0		
	K4	0	0	0	0	0		0		
	K5	0	0	0	0	0		0		
	K6	0	0	0	0	0		0		
	K7	0	0	0	0	0		0		
	K8	0	0	0	0	0		0		
T3	K1	1	1	1	1	0			0.7	
	K2	0	0	0	1	1			0.3	
	K3	0	0	0	0	0			0	
	K4	0	0	0	0	0			0	
	K5	0	0	0	0	0			0	
	K6	0	0	0	0	0			0	
	K7	0	0	0	0	0			0	
	K8	0	0	0	0	0			0	
T4	K1	1	1	1	1	1				0.8
	K2	0	0	0	1	1				0.2
	K3	0	0	0	0	0				0
	K4	0	0	0	0	0				0
	K5	0	0	0	0	0				0
	K6	0	0	0	0	0				0
	K7	0	0	0	0	0				0
	K8	0	0	0	0	0				0
T5	K1	1	1	1	1	1			0.8	0.74
	K2	0	0	0	1	1			0.2	0.26
	K3	0	0	0	0	0			0	0
	K4	0	0	0	0	0			0	0
	K5	0	0	0	0	0			0	0
	K6	0	0	0	0	0			0	0
	K7	0	0	0	0	0			0	0
	K8	0	0	0	0	0			0	0

## Data Availability

No new data were created or analyzed in this study. Data sharing is not applicable to this article.
